# Three new species of *Axinulus* (Bivalvia: Thyasiridae) from the Japan and Kuril-Kamchatka trenches and abyssal zone of the northern Pacific Ocean

**DOI:** 10.7717/peerj.15543

**Published:** 2023-06-14

**Authors:** Gennady M. Kamenev

**Affiliations:** A.V. Zhirmunsky National Scientific Center of Marine Biology, Far Eastern Branch, Russian Academy of Sciences, Vladivostok, Russia

**Keywords:** Bivalvia, Taxonomy, Northern Pacific, Oceanic trenches

## Abstract

The Thyasiridae is one of the most species-rich families of bivalves in the deep-sea areas of the northern Pacific Ocean. Many thyasirid species form abundant populations in these regions and play an important role in the functioning of deep-sea benthic communities. However, most of these deep-sea thyasirid species have not been identified and many of them are new to science. Based on the material of bivalves collected by eight deep-sea expeditions in the northern Pacific Ocean during the period from 1954 to 2016, three new species of the genus *Axinulus* (*Axinulus krylovae* sp. nov., *A. alatus* sp. nov., and *A. cristatus* sp. nov.) are described from the Kuril-Kamchatka and Japan trenches, the Bering Sea, and other deep-water regions of the northern Pacific Ocean (3,200–9,583 m depth). The new species are distinguished due to a unique and complex sculpture of the prodissoconch, including tubercles and numerous thin folds of varying length and shape, as well as due to a thickening of the shell in the adductor scar areas, thus rendering the scars raised above the inner surface of the shell. Comparisons with all species of the genus *Axinulus* are provided.

## Introduction

In recent years, international expeditions organized by the Russian Federation and Germany have sampled the deep-sea benthic fauna in an extensive region of the northwestern Pacific Ocean with depths greater than 3,000 m. The studies were focused on the composition and distribution of the benthic fauna in the deep-sea basins of the Seas of Japan and Okhotsk, the Kuril-Kamchatka Trench, and at the abyssal plain of the Pacific Ocean adjacent to the Kuril-Kamchatka Trench (Malyutina & Brandt, 2013; Brandt & Malyutina, 2015; Malyutina, Chernyshev & Brandt, 2018; Brandt et al., 2019). Benthic macrofauna rich in species number and abundance was found in these deep-sea ecosystems, with bivalves being one of the dominant groups of animals (Brandt et al., 2013, 2015, 2018, 2020; Kamenev, 2013, 2014, 2015, 2018a, 2018b, 2018c, 2019; Kamenev et al., 2022). Among the bivalve fauna of all the studied deep-sea areas of the northwestern Pacific Ocean, the Thyasiridae was the most species-rich family and many thyasirids were the dominant species in terms of abundance in the benthic macrofaunal communities (Kamenev, 2013, 2015, 2018a, 2019; Kamenev et al., 2022). At least 14 thyasirid species were found at depths greater than 3,000 m in the abyssal and hadal zones of the northwestern Pacific Ocean. They have not been identified to the species level and are most likely new to science. Moreover, previous studies of the northwestern Pacific deep-sea fauna revealed many species of thyasirids in various oceanic trenches (Belyaev & Mironov, 1977; Belyaev, 1989; Okutani, Fujikura & Kojima, 1999; Allen, 2015), most of them remain so far unidentified to the species level (Belyaev, 1989). Thus, despite the great importance of thyasirids in the functioning of the deep-sea ecosystems of the northwestern Pacific Ocean, the abyssal and hadal thyasirid fauna in the region is almost not studied, except three species (*Axinulus hadalis* (Okutani, Fujikura & Kojima, 1999), *Axinulus philippinensis* Allen, 2015, and *Thyasira kaireiae* (Okutani, Fujikura & Kojima, 1999)), which were found in the Japanese and Philippine trenches (Okutani, Fujikura & Kojima, 1999; Allen, 2015).

Several years ago, the author of this article started a study of unidentified deep-sea species of the Thyasiridae found in the northwestern Pacific Ocean. As a result of this work, six new species have been described from the abyssal and hadal zones of the Sea of Okhotsk and the Bering Sea, the Kuril-Kamchatka and Japan trenches, as well as the oceanic plain adjacent to these trenches (Kamenev, 2020, 2023). The present article is a continuation of the study of northwestern Pacific deep-sea thyasirids and presents a description of three new species of the genus *Axinulus* that were found in the Japan and Kuril-Kamchatka trenches, as well as in the abyssal zone of other deep-sea regions of the northern Pacific Ocean.

## Materials and Methods

### Material studied

The material examined in this study was collected from 1954 to 1990 by expeditions of the P.P. Shirshov Institute of Oceanology, Russian Academy of Sciences, Moscow (IO RAS) in the hadal zone of the Kuril-Kamchatka Trench and from the Pacific abyssal plain adjacent to the Kuril-Kamchatka Trench (RV *Vityaz*, cruise no. 19, August 17–October 29, 1954; RV *Vityaz*, cruise no. 39, July 7–September 13, 1966), in the hadal zone of the Japan Trench (RV *Vityaz*, cruise no. 59, May 26–July 5, 1976), in the Gulf of Alaska (RV *Vityaz*, cruise no. 45, April 23–July 10, 1969), and from the bottom of the Commander Basin (Bering Sea) (RV *Akademik Mstislav Keldysh*, cruise no. 22, July 25–October 27, 1990), as well as by the German-Russian deep-sea expeditions KuramBio (RV *Sonne*, cruise no. 223, July 21–September 7, 2012) and KuramBio II (RV *Sonne*, cruise no. 250, August 16–September 29, 2016) from the Pacific abyssal plain adjacent to the Kuril-Kamchatka Trench and in the hadal zone of the Kuril-Kamchatka Trench; and by the Russian-German deep-sea expedition SokhoBio (RV *Akademik M.A. Lavrentyev*, cruise no. 71, July 6–August 6, 2015) in the abyssal zone of the Pacific slope of the Kuril Islands. The new species of *Axinulus* were found among the 72 samples of benthic fauna at depths of 3,200–9,583 m. The methods for the sample collecting and fixing have been in detail described previosly (Monin, 1983; Brandt & Malyutina, 2015; Malyutina, Chernyshev & Brandt, 2018; Brandt et al., 2020; Kamenev, 2020, 2023). The samples obtained during the expeditions of the IO RAS were stored in the IO RAS Ocean Benthic Fauna collection. The samples collected by the KuramBio, KuramBio II, and SokhoBio expeditions were stored at the Museum (MIMB) of A.V. Zhirmunsky National Scientific Center of Marine Biology, Far Eastern Branch, Russian Academy of Sciences (NSCMB FEB RAS), Vladivostok, Russia. The type and other materials of the new species were deposited at the MIMB, IO RAS, and Senckenberg Museum Frankfurt, Germany (SMF).

Additional material was also examined for the comparison purposes: *Axinulus careyi* Bernard, 1979 (holotype, LACM 1990 (Natural History Museum of Los Angeles County, Los Angeles, CA, USA)); paratype, SBNHM 55440 (Santa Barbara of the Natural History Museum, Santa Barbara); *Axinulus kelliaeformis* Okutani, 1962 (holotype, NSMT Mo 69697 (National Museum of Nature and Science, Tsukuba, Japan)); *Axinulus thackergeigeri* Valentich-Scott & Coan, 2012 in Coan & Valentich-Scott, 2012 (holotype, SBNHM 149742; paratype, SBNHM 149743); *Axinulus obliquus* Okutani, 1968 (holotype, NSMT Mo 69719, and photos of paratype and additional specimens, NSMT Mo 69720 (photos by Dr. H. Saito)); *Clausina croulinensis* Jeffreys, 1847 (neotype, USNM 62048 (National Museum of Natural History, Washington, D.C., USA), photos from USNM Web site); *Cryptodon (Axinulus) brevis* Verrill & Bush, 1898 (holotype, USNM 159873, photos from USNM Web site); *Cryptodon* (*Axinulus) simplex* Verrill & Bush, 1898 (holotype, USNM 159888, photos from USNM Web site); *Cryptodon equalis* Verrill & Bush, 1898 (holotype, USNM 74302, photos from USNM Web site); *Maorithyas hadalis* Okutani, Fujikura & Kojima, 1999 (paratype, NSMT Paratype C Mo 71432-c). Information and photos from USNM Web site provided with the permission of the National Museum of Natural History, Smithsonian Institution, 10th and Constitution Ave. N.W., Washington, DC 20560-0193 (http://www.nmnh.si.edu/).

## Methods

**Figure 1:**
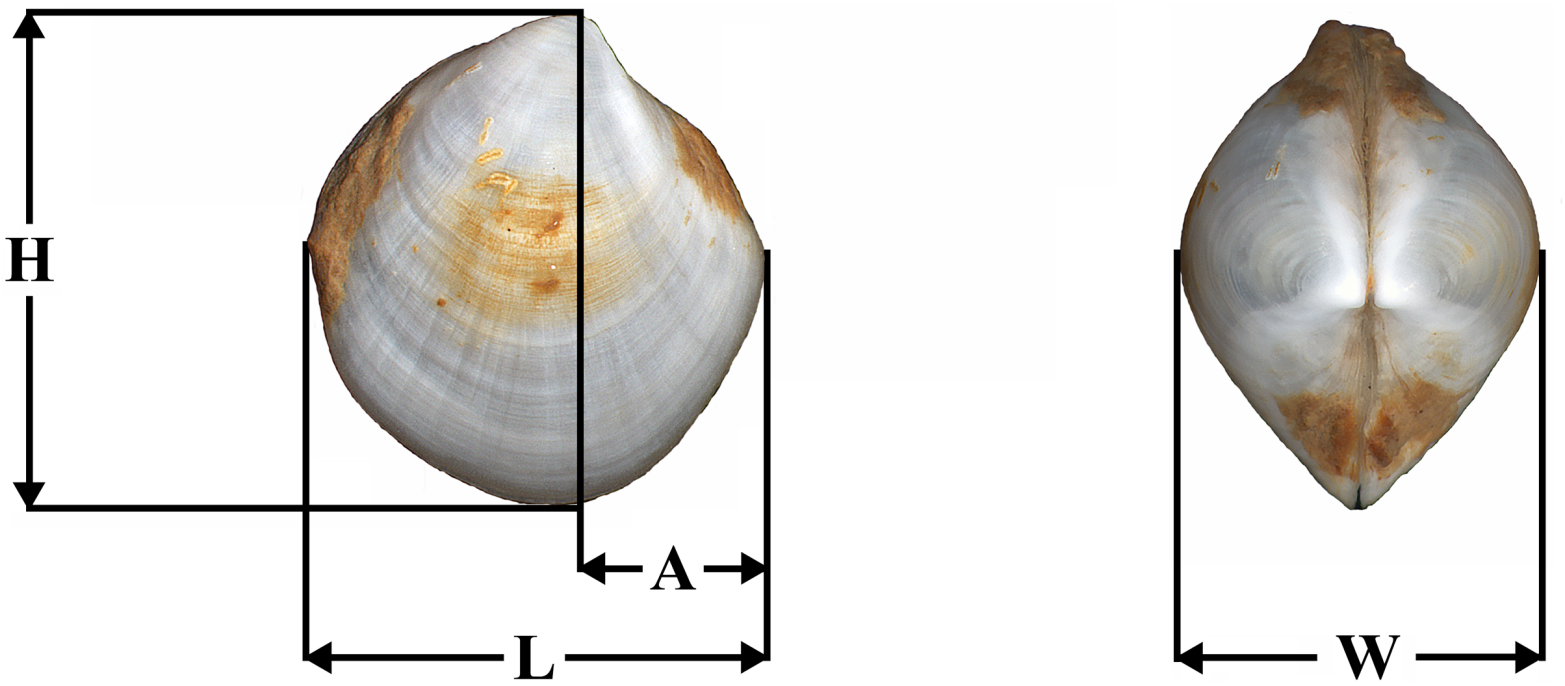
Placement of shell measurements. Abbreviations: L, shell length; H, height; A, anterior end length; W, shell width.

Shell length (L), height (H), anterior end length (A), and shell width (W) were measured with the ocular micrometer at the accuracy level of 0.1 mm (Fig. 1). The ratios H/L, A/L, and W/L were determined.

The methods for the sample preparation and the shell morphology study using the scanning microscopy as well as the gross anatomy analysis of the preserved alive-taken specimens have been described by Kamenev (2020, 2023). The microscopic studies of the new species described here were carried out at the Far Eastern Center of Electron Microscopy of the NSCMB FEB RAS. To describe the shell morphology and body anatomy of the new species belonging to the genus *Axinulus*, the terms proposed by Payne & Allen (1991) and Oliver & Killeen (2002) were used.

### Nomenclatural acts

The electronic version of this article in Portable Document Format (PDF) will represent a published work according to the International Commission on Zoological Nomenclature (ICZN), and hence the new names contained in the electronic version are effectively published under that Code from the electronic edition alone. This published work and the nomenclatural acts it contains have been registered in ZooBank, the online registration system for the ICZN. The ZooBank LSIDs (Life Science Identifiers) can be resolved and the associated information viewed through any standard web browser by appending the LSID to the prefix http://zoobank.org/. The LSID for this publication is: urn:lsid:zoobank.org:pub:0AE8C4E1-0F13-478E-81E9-287980139049. The online version of this work is archived and available from the following digital repositories: PeerJ, PubMed Central SCIE and CLOCKSS.

## Results

### Systematics

**Class** Bivalvia

**Order** Lucinoida Gray, 1854

**Superfamily** Thyasiroidea Dall, 1900 (1895)

**Family** Thyasiridae Dall, 1900 (1895)

**Genus** *Axinulus* Verrill & Bush, 1898

Type species (by original designation): *Cryptodon (Axinulus) brevis* Verrill & Bush, 1898

**Diagnosis (after Kamenev, 2020):** Shell small (<10 mm), equilateral or subequilateral, ovate to ovate-rhomboidal, slightly higher than long; margins entire, without any posterior sinus, but sometimes with weak angulation of posterior margin; posterior sulcus absent or weak. Lunule variably expressed, indistinct to deep; escutcheon and auricle sometimes present. Hinge plate edentulous, sometimes with a small swelling. Ligament sunken, sometimes visible externally. Ctenidium of a single demibranch, foot vermiform, lateral body pouches without or with distinct lobes.

**Remarks:** Despite that the WoRMS (WoRMS Editorial Board, 2023) currently lists 13 species of the genus *Axinulus*, some authors (Zelaya, 2010; Allen, 2015; Oliver, 2015; Kamenev, 2020) recognize only eight species as belonging to the genus (*Axinulus alleni* (Carrozza, 1981), *Axinulus brevis* (Verrill & Bush, 1898), *Axinulus croulinensis* (Jeffreys, 1847), *Axinulus antarcticus* Zelaya, 2010, *A. philippinensis*, *Axinulus subequatorius* (Payne & Allen, 1991), *Axinulus oliveri* Kamenev, 2020, and *Axinulus roseus* Kamenev, 2020). Therefore, the new species described herein were compared only with the above species. Assignment the other five species to the genus *Axinulus* is doubtful (Oliver, 2015; Kamenev, 2020).

**Figure 2:**
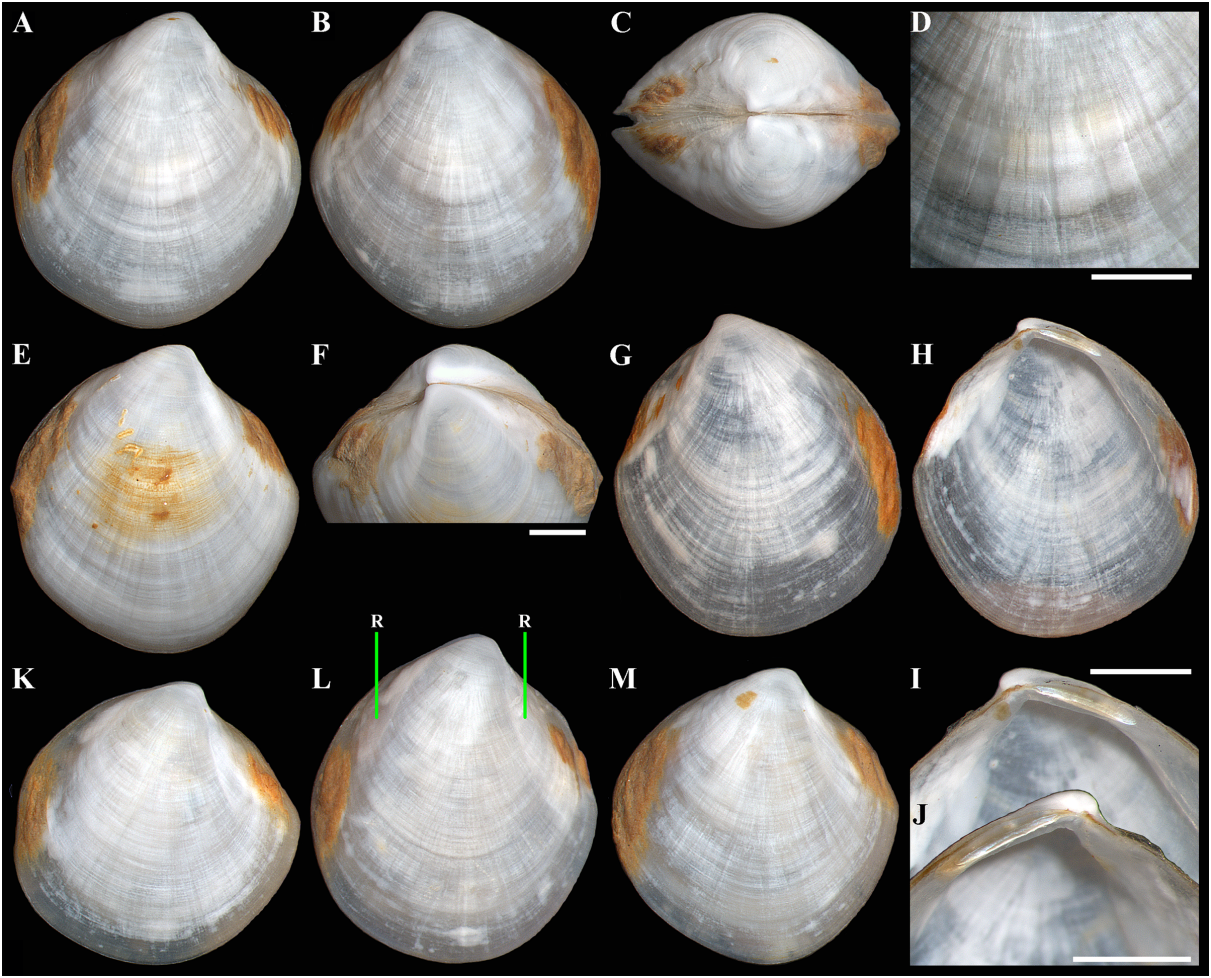
*Axinulus krylovae* sp. nov. (A–D) Holotype (IO RAS BIV00984), exterior and dorsal views, and sculpture of central shell part, shell length 4.9 mm. (E and F) Paratype (IO RAS BIV00985), exterior and oblique dorsal views, shell length 5.0 mm. (G–J) Exterior and interior views of right and left valves, and hinge plates of both valves, valves length 4.4 mm. (K–M) Variability of shell shape, exterior view of right valves of specimens from holotype locality: (K) shell length 3.5 mm ; (L) shell length 4.3 mm; (M) shell length 4.0 mm. Abbreviation: R, radial, whitish ray. Scale bar: D, F, I, J = 1 mm.

**Figure 3:**
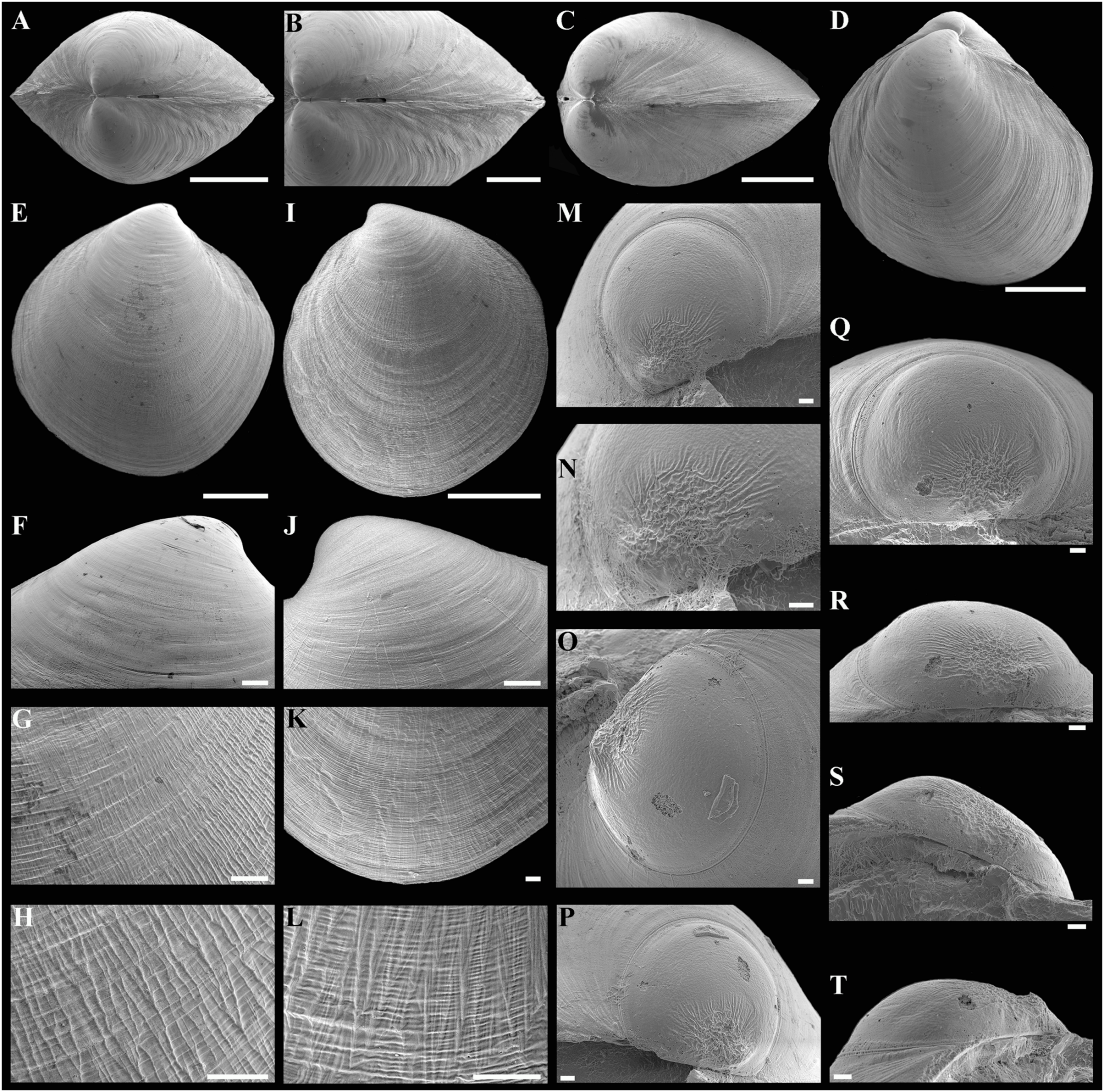
Scanning electron micrographs of *Axinulus krylovae* sp. nov. (A) Dorsal view of both valves. (B) Dorsal view of posterodorsal margin. (C) Lunule. (D) Oblique dorsal view of lunule. (E–H) Specimen from the Japan Trench, depth 7,540 m: (E) Exterior view of right valve. (F) Sculpture of beak region. (G and H) Sculpture of central shell part. (I–L) Specimen from the Kuril-Kamchatka Trench, depth 8,734 m: (I) Exterior view of right valve. (J) Sculpture of beak region. (K) Sculpture of ventral shell part. (L) Sculpture of central shell part. (M–P) Prodissoconch of specimen from the Japan Trench. (Q–T) Prodissoconch of specimen from the Kuril-Kamchatka Trench. Scale bars: A, C–E, I = 1 mm; B = 500 μm; F–H, J–L, = 100 µm; M–T = 10 µm.

**Figure 4:**
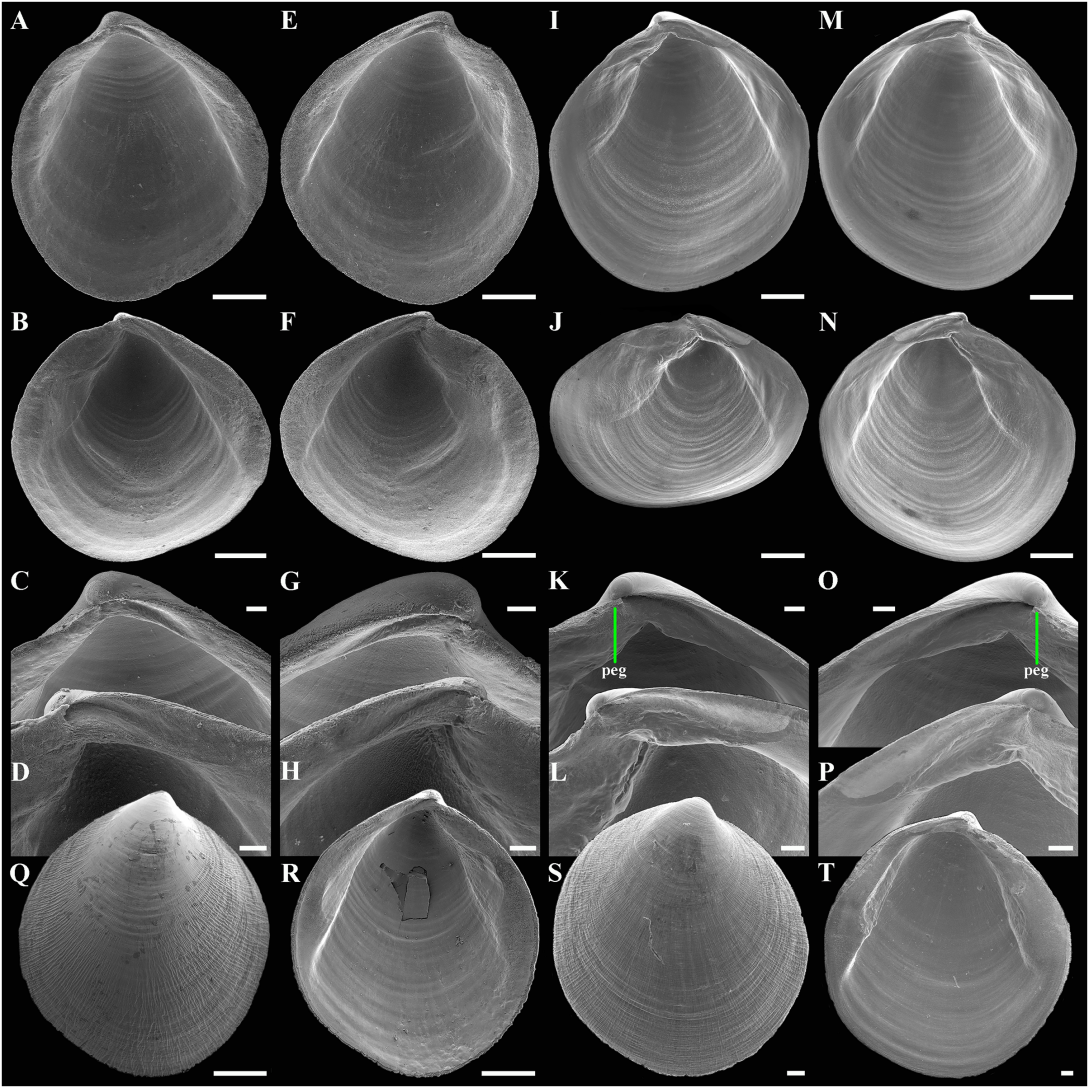
Scanning electron micrographs of *Axinulus krylovae* sp. nov. (A–H) Specimen from the Kuril-Kamchatka Trench, depth 8,700 m: (A and B) Interior and ventral views of right valve. (C and D) Hinge plate and ligamental groove of right valve. (E and F) Interior and ventral views of left valve. (G and H) Hinge plate and ligamental groove of left valve. (I–P) Specimen from the Japan Trench, depth 7,540 m: (I and J) Interior and ventral views of right valve. (K and L) Hinge plate and ligamental groove of right valve. (M and N) Interior and ventral views of left valve. (O and P) Hinge plate and ligamental groove of left valve. (Q–T) Exterior and interior views of valves of young specimens: (Q and R) Specimen from the Kuril-Kamchatka Trench, depth 8,700 m. (S and T) Specimen from the abyssal plain adjacent to the Kuril-Kamchatka Trench, depth 5,125 m. Scale bars: A, B, E, F, I, J, M, N, Q, R = 500 µm; C, D, G, H, K, L, O, P, S, T = 100 µm.

**Figure 5:**
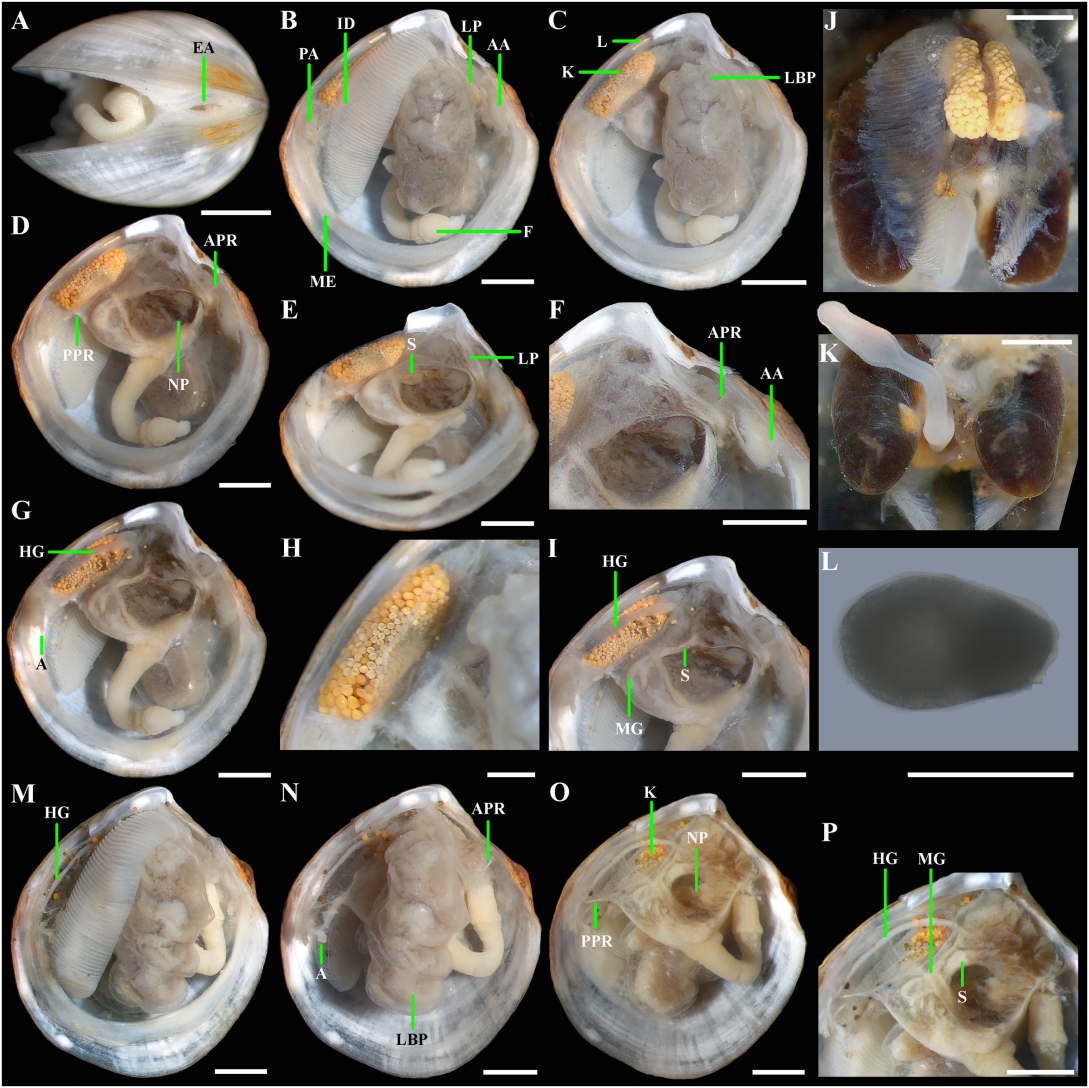
*Axinulus krylovae* sp. nov. (A) Exhalant aperture. (B) Gross anatomy after removal of right valve and mantle. (C) Gross anatomy after further removal of right ctenidium. (D and E) Gross anatomy after further removal of right lateral body pouch. (F) Labial palps, anterior pedal and adductor muscles. (G) Gross anatomy after further removal of right kidney. (H) Kidney with orange granules. (I) Digestive system. (J) Posterior view of kidneys, ctenidium, and lateral body pouches of live specimen after removal shell and mantle. (K) Foot of live specimen. (L) Egg (photo by Dr. O.V. Yurchenko (NSCMB FEB RAS)). (M) Gross anatomy after removal of right valve, mantle, right kidney, and posterior adductor muscle. (N) Gross anatomy after further removal of right ctenidium and mantle edge. (O) Gross anatomy after further removal of right lateral body pouch and anterior adductor muscle. (P) Digestive system. Abbreviations: AA, anterior adductor muscle; APR, anterior pedal retractor muscle; EA, exhalant aperture; F, foot; HG, hind gut; ID, inner demibranch; K, kidney; L, ligament; LBP, lateral body pouch; LP, labial palps; ME, mantle edge; MG, mid gut; NP, neck of lateral body pouch; PA, posterior adductor muscle; PPR, posterior pedal retractor muscle; S, stomach. Scale bars: A–K, M–P = 1 mm; L = 100 µm.

***Axinulus krylovae* sp. nov.**

(Figs. 2–5, Tables 1 and 2)

**Table 1:** Additional material of *Axinulus krylovae* sp. nov. examined in the present study.

Ship, cruise no.	Station	Date	Start		End		Depth (m)	Gear	*N*	Depository
			Latitude °N	Longitude °E	Latitude °N	Longitude °E				
**Japan Trench**										
*Vityaz* 45	6151	28.06.1969	37°41.5′	143°54.3′	–	–	7,370	ST	1	IORAS BIV00980
*Vityaz* 59	7500	21.06.1976	37°38′	143°58′	–	–	7,370–7,350	GT	1	IORAS BIV00992
	7503	23.06.1976	36°44′	143°19′	–	–	7,540	GT	74	IORAS BIV00986
	7511	27.06.1976	38°38′	144°06′	–	–	7,500	OG	1	IORAS BIV00987
**Kuril-Kamchatka Trench**										
*Vityaz* 19	3168	04.10.1954	45°41.7′	152°36.7′	–	–	6,150	OG	8	IORAS BIV00981
*Vityaz* 39	5615	03.08.1966	45°56′	153°28′	–	–	8,060–8,135	ST	2	IORAS BIV00982
*Sonne* 250	17	22.08.2016	45°51.514′	153°50.581′	45°51.401′	153°50.406′	8,183–8,184	EBS	6	MIMB 45900
	19	23.08.2016	45°51.566′	153°50.451′	45°51.412′	153°50.215′	8,189–8,187	EBS	2	MIMB 45901
	25	25.08.2016	45°55.235′	152°47.464′	–	–	6,068	GKG	3	MIMB 45902
	28	26.08.2016	45°53.959′	152°45.838′	45°54.520′	152°47.204′	6,087–6,047	EBS	1	MIMB 45903
	29	26.08.2016	45°56.587′	152°54.251′	45°56.570′	152°54.499′	6,204–6,202	AGT	2	MIMB 45904
	30	27.08.2016	45°56.821′	152°51.185′	45°56.834′	152°50.943′	6,168–6,164	EBS	8	MIMB 45905
	37	28.08.2016	45°38.604′	152°55.911′	–	–	7,136	GKG	1	MIMB 45906
	40	29.08.2016	45°40.686′	152°57.374′	45°40.839′	152°57.687′	7,060–7,055	EBS	1	MIMB 45907
	42	30.08.2016	45°40.173′	152°57.457′	45°40.263′	152°57.638′	7,122–7,120	EBS	6	MIMB 45908
	49	04.09.2016	45°28.752′	153°11.649′	–	–	8,739	GKG	3	MIMB 45909
	51	05.09.2016	45°28.748′	153°11.654′	–	–	8,737	MUC	1	MIMB 45910
	52	05.09.2016	45°29.347′	153°11.415′	45°29.187′	153°11.138′	8,738–8,699	EBS	24	MIMB 45911
	54	06.09.2016	45°28.139′	153°10.624′	45°28.125′	153°10.109′	8,701–8,735	AGT	14	MIMB 45912
	55	07.09.2016	45°29.491′	153°12.540′	45°29.580′	153°12.240′	8,740–8,735	EBS	11	MIMB 45913
	67	10.09.2016	45°12.944′	152°42.844′	–	–	9,495	GKG	1	MIMB 45914
	75	12.09.2016	44°39.883′	151°28.136′	–	–	8,221	GKG	9	MIMB 45915
	77	13.09.2016	45°13.892′	152°50.774′	45°14.219′	152°49.956′	9,577–9,583	EBS	11	MIMB 45916
	89	16.09.2016	44°39.325′	151°27.340′	44°39.053′	151°27.343′	8,215–8,217	EBS	8	MIMB 45917
	90	17.09.2016	44°41.759′	151°26.554′	44°41.992′	151°26.321′	8,271–8,273	AGT	24	MIMB 45918
	94	18.09.2016	44°06.852′	151°25.539′	–	–	6,531	GKG	11	MIMB 45919
	97	18.09.2016	44°06.668′	151°24.878′	44°06.942′	151°24.888′	6,551–6,561	EBS	13	MIMB 45920
	98	19.09.2016	44°06.152′	151°25.705′	44°06.253′	151°25.935′	6,441–6,442	AGT	12	MIMB 45921
	100	20.09.2016	44°12.378′	150°39.053′	–	–	9,305	GKG	3	MIMB 45922
	103	21.09.2016	44°12.499′	150°39.055′	44°12.502′	150°37.258′	9,301–9,431	AGT	1	MIMB 45923
	105	22.09.2016	44°12.391′	150°36.006′			9,540	GKG	1	MIMB 45924
**Kuril-Kamchatka Trench area and abyssal plain adjacent to trench (depth less than 6,000 m)**										
*Vityaz* 19	3102	22.08.1954	41°16′	147°27.7′	–	–	5,210	OG	1	IORAS BIV00983
*Sonne* 223	1-5	29.07.2012	43°58.17′	157°19.88′	–	–	5,410	GKG	1	MIMB 45925
	2-5	02.08.2012	46°13.99′	155°33.10′	–	–	4,869	GKG	1	MIMB 45926
	2-9	02.08.2012	46°13.60′	155°33.42′	46°14.93′	155°32.57′	4,866–4,860	EBS	3	MIMB 45927
	2-11	03.08.2012	46°13.69′	155°33.29′	46°14.87′	155°32.49′	4,869–4,861	AGT	5	MIMB 45928
	3-4	04.08.2012	47°14.32′	154°42.26′	–	–	4,982	GKG	6	MIMB 45929
	3-9	05.08.2012	47°13.83′	154°41.88′	47°14.87′	154°43.18′	4,988–4,998	EBS	3	MIMB 45930
	4-4	07.08.2012	46°57.97′	154°32.49′	–	–	5,766	GKG	3	MIMB 45931
	5-4	10.08.2012	43°35.01′	153°58.09′	–	–	5,379	GKG	1	MIMB 45932
	6-4	13.08.2012	42°28.98′	153°59.97′	–	–	5,297	GKG	1	MIMB 45933
	7-4	16.08.2012	43°02.31′	152°59.16′	–	–	5,222	GKG	7	MIMB 45934
	7-5	16.08.2012	43°02.24′	152°59.09′	–	–	5,223	GKG	3	MIMB 45935
	7-9	17.08.2012	43°02.87′	152°59.45′	43°01.50′	152°58.35′	5,216–5,221	EBS	3	MIMB 45936
	7-10	17.08.2012	43°02.78′	152°59.30′	43°01.65′	152°58.45′	5,217–5,223	EBS	20	MIMB 45937
	8-4	19.08.2012	42°14.57′	151°43.51′	–	–	5,130	GKG	1	MIMB 45938
	8-5	19.08.2012	42°14.57′	151°43.51′	–	–	5,130	GKG	5	MIMB 45939
	8-9	20.08.2012	42°14.69′	151°44.05′	42°14.26′	151°42.49′	5,127	EBS	18	MIMB 45940
	8-12	21.08.2012	42°14.73′	151°44.38′	42°14.32′	151°42.94′	5,112–5,126	EBS	7	MIMB 45941
	9-5	23.08.2012	40°34.96′	151°00.07′	–	–	5,401	GKG	1	MIMB 45942
	9-9	23.08.2012	40°35.49′	150°59.92′	40°34.25′	150°59.91′	5,399–5,398	EBS	2	MIMB 45943
	9-10	24.08.2012	40°36.13′	151°00.07′	40°35.31′	151°00.12′	5,406–5,404	AGT	3	MIMB 45944
	9-12	25.08.2012	40°35.40′	150°59.84′	40°34.27′	150°59.00′	5,398	EBS	1	MIMB 45945
	10-5	26.08.2012	41°11.99′	150°05.75′	–	–	5,251	GKG	1	MIMB 45946
	10-12	27.08.2012	41°11.70′	150°05.56′	41°13.03′	150°05.71′	5,250–5,255	EBS	4	MIMB 45947
	11-4	29.08.2012	40°12.86′	148°05.92′	–	–	5,348	GKG	5	MIMB 45948
	11-9	30.08.2012	40°13.26′	148°06.24′	40°12.37′	148°05.43′	5,348–5,350	EBS	5	MIMB 45949
	11-11	30.08.2012	40°13.55′	148°06.77′	40°12.90′	148°06.20′	5,349–5,352	AGT	1	MIMB 45950
	11-12	31.08.2012	40°13.10′	148°06.45′	40°12.10′	148°05.53′	5,351–5,348	EBS	6	MIMB 45951
	12-2	31.08.2012	39°43.43′	147°09.98′	–	–	5,243	GKG	7	MIMB 45952
	12-4	01.09.2012	39°43.80′	147°10.16′	39°42.49′	147°09.37′	5,224–5,215	EBS	3	MIMB 45953
*Sonne* 250	6	18.08.2016	43°49.197′	151°45,609′	–	–	5,497	GKG	1	MIMB 45954
	8	19.08.2016	43°48.593′	151°46.433′	43°48.598′	151°46.477′	5,107	EBS	13	MIMB 45955
	10	20.08.2016	43°48.602′	151°47.124′	43°48.455′	151°47.171′	5,352–5,104	EBS	7	MIMB 45956
	61	08.09.2016	45°09.997′	153°45.419′	–	–	5,741	GKG	5	MIMB 45957
	65	09.09.2016	45°10.226′	153°44.178′	45°10.160′	153°44.052′	5,734–5,752	EBS	1	MIMB 45958
	86	15.09.2016	45°01.202′	151°06.008′	45°01.371′	151°06.001′	5,572–5,530	AGT	19	MIMB 45959
	87	16.09.2016	45°01.383′	151°05.527′	45°01.651′	151°05.522′	5,496–5,478	EBS	1	MIMB 45960

**Note:**

GT, Galathea trawl; OG, Okean grab (0.25 m^2^); ST, Sigsbee trawl; GKG, giant box corer (0.25 m^2^); EBS, epibenthic sledge; AGT, Agassiz trawl; *N*, number of live specimens.

**Table 2:** *Axinulus krylovae* sp. nov. Shell measurements (mm), indices and summary statistics of indices.

Depository	*L*	*H*	*A*	*W*	*H/L*	*A/L*	*W/L*
Paratype IORAS BIV00985	5.1	5.5	2.3	3.5	1.078	0.451	0.686
Paratype IORAS BIV00985	5.0	5.4	1.9	3.2	1.080	0.380	0.640
Holotype IORAS BIV00984	4.9	5.3	2.0	3.4	1.082	0.408	0.694
IORAS BIV00986	4.8	5.5	2.2	3.3	1.146	0.458	0.688
IORAS BIV00986	4.8	5.2	1.8	3.1	1.083	0.375	0.646
IORAS BIV00986	4.7	4.8	2.1	3.0	1.021	0.447	0.638
Paratype MIMB 45898	4.7	5.3	1.8	3.6	1.128	0.383	0.766
IORAS BIV00986	4.6	4.6	1.7	2.9	1.000	0.370	0.630
Paratype IORAS BIV00985	4.5	5.1	1.9	3.2	1.133	0.422	0.711
Paratype IORAS BIV00985	4.5	4.8	1.7	3.1	1.067	0.378	0.689
IORAS BIV00986	4.3	4.5	1.9	3.1	1.047	0.442	0.721
Paratype MIMB 45899	4.2	4.7	1.9	3.0	1.119	0.452	0.714
Paratype SMF 372429	4.1	4.3	1.6	2.8	1.049	0.390	0.683
IORAS BIV00986	4.2	5.0	1.8	3.0	1.190	0.429	0.714
IORAS BIV00986	4.2	4.4	1.5	3.0	1.048	0.357	0.714
IORAS BIV00986	4.2	4.6	2.0	2.9	1.095	0.476	0.690
IORAS BIV00986	4.1	4.4	1.6	2.8	1.073	0.390	0.683
IORAS BIV00986	4.0	4.1	1.6	2.4	1.025	0.400	0.600
IORAS BIV00986	3.9	4.1	1.5	2.5	1.051	0.385	0.641
IORAS BIV00986	3.8	4.3	1.7	2.6	1.132	0.447	0.684
IORAS BIV00986	3.8	4.0	1.8	2.4	1.053	0.474	0.632
IORAS BIV00986	3.7	3.8	1.3	2.4	1.027	0.351	0.649
IORAS BIV00986	3.7	3.9	1.5	2.4	1.054	0.405	0.649
IORAS BIV00986	3.6	4.1	1.6	2.4	1.139	0.444	0.667
IORAS BIV00986	3.6	3.8	1.4	2.4	1.056	0.389	0.667
IORAS BIV00986	3.5	3.7	1.5	2.3	1.057	0.429	0.657
IORAS BIV00986	3.5	3.5	1.5	2.2	1.000	0.429	0.629
IORAS BIV00986	3.5	3.8	1.4	2.3	1.086	0.400	0.657
IORAS BIV00986	3.4	3.7	1.4	2.1	1.088	0.412	0.618
IORAS BIV00986	3.3	3.7	1.6	2.2	1.121	0.485	0.667
IORAS BIV00986	3.3	3.7	1.5	2.2	1.121	0.455	0.667
IORAS BIV00986	2.4	2.9	0.9	1.6	1.208	0.375	0.667
Statistics	*L*	*H*	*A*	*W*	*H/L*	*A/L*	*W/L*
Mean	–	–	–	–	1.083	0.415	0.671
SE	–	–	–	–	0.039	0.032	0.028
SD	–	–	–	–	0.050	0.037	0.035
Min	–	–	–	–	1.000	0.351	0.600
Max	–	–	–	–	1.208	0.485	0.766

**Note:**

*L*, shell length; *H*, height; *A*, anterior end length; *W*, width.

*Axinulus* sp.: Kamenev, 2015, p. 191.

“*Genaxinus*” sp. 1: Kamenev, 2019, p. 6, 7.

urn:lsid:zoobank.org:act:F3D588B6-84FD-44A7-99BF-7FB53AC893F3

**Type material and locality:** Holotype (IORAS BIV00984), Japan Trench, Pacific Ocean (36°44′N, 143°19′E), 7,540 m, Galathea trawl, Coll. F.A. Pasternak, 23-VI-1976 (RV *Vityaz*, cruise no. 59, stn. 7503); paratypes (4) (IORAS BIV00985) and paratype (MIMB 45898) from holotype locality; paratype (MIMB 45899) and paratype (SMF 372429), Kuril-Kamchatka Trench, Pacific Ocean (45°56.821′N, 152°51.185′E–45°56.834′N, 152°50.943′E), 6,168–6,164 m, epibenthic sledge, Coll. A. Brandt, 27-VIII-2016 (RV *Sonne*, cruise no. 250, stn. 30).

**Other material examined:** 438 live specimens (Table 1).

**Diagnosis:** Shell medium in size (to 5.2 mm in length), elongated ovate to rhomboidal, with two radiating, whitish, elongated triangular rays extending from beaks to anteroventral and posteroventral margins. Sculpture of closely spaced commarginal riblets forming weak undulations and conspicuous, closely spaced, radial ribs and rays as slightly convex overlap areas of varying width over commarginal riblets. Posterior folds and sulcus absent. Escutcheon and auricle absent. Lunule as a crest, raised, wide, weakly defined. Ligament sunken, only slightly visible externally, long. Prodissoconch small (length 131–158 µm), irregularly convex, with distinct tubercle in anterior part; initial part densely sculptured, chaotically arranged, short, curved plicae and numerous, radial, thin folds. Adductor muscle scars distinct, outline elongate-triangular, raised above inner shell surface because of thickening of shell. Lateral body pouches large, simple, without numerous distinct lobes.

**Description.** Shell medium in size (to 5.2 mm in length and 5.7 mm in height), strongly inflated (W/L = 0.671 ± 0.028), slightly higher than long (H/L = 1.083 ± 0.039); elongated ovate to rhomboidal, equivalve, subequilateral, white, thick, with two curved, radial, whitish, elongated triangular, opaque rays extending from beaks to anteroventral and posteroventral margins, respectively, formed by internal thickening shell; patches of silty deposit adhering to anteriodorsal and posterior shell margins (Fig. 2 and Table 2). Periostracum thin, colorless, translucent, adherent. Dissoconch sculptured with thin, closely spaced, commarginal riblets forming weak, narrow, irregular, undulations and conspicuous, closely spaced, radial ribs and rays with overlap areas of varying width over commarginal riblets. Beaks small, raised, prosogyrate, curved slightly inwards, slightly anterior to midline (A/L = 0.415 ± 0.032) (Fig. 3 and Table 2). Anterodorsal shell margin convex, steeply sloping from beaks, forming a rounded angle at transition to anterior margin. Anterior margin curved, smoothly transitioning to ventral margin. Ventral margin strongly curved, sometimes slightly angulate. Posterodorsal margin slightly convex, steeply sloping from beaks, smoothly transitioning to posterior margin. Posterior margin slightly curved or straight, smoothly transitioning to ventral margin. Posterior folds and sulcus absent but posterior shell area a little flattened. Escutcheon and auricle absent. Lunule as a crest, raised, long, wide, weakly defined, demarcated by weak, long, rounded ridges along entire anterodorsal shell margin (Figs. 3C and 3D). Ligament opisthodetic, sunken, slightly visible externally as a narrow strip between valves, thick, almost straight, long, about half the length of posterodorsal shell margin, lying in deep, slightly curved, wide groove at surface of hinge plate (Fig. 4). Prodissoconch small (length 131–158 µm), ovate in outline, slightly drawn out anteriorly, irregularly convex, flattened anteriorly, with a distinct tubercle and shallow depression in initial part (Figs. 3M–3T). Tubercle and depression of prodissoconch sculptured with numerous, densely spaced, short and chaotically arranged folds, forming a rounded area; numerous short, thin, radial folds of about same length radiating from this area; remaining surface of prodissoconch smooth or slightly bumpy (Fig. 3N). Hinge plate thickened, with a small peg under beak in each valve and long, deep ligamental groove (Fig. 4). Adductor muscle scars distinct, long, elongated triangular in outline, extending into umbonal cavity, bearing thin, indistinct, radial striation; shell strongly thickened in scar area, thus rendering muscle scars raised above inner shell surface. Posterior adductor scar narrow, straight; anterior adductor scar curved, approximately twice as wide as posterior (Fig. 4).

*Gross anatomy:* Mantle thin; margins thickened and unfused except limited interconnection at the posterior ventral margin with a small exhalant aperture below the posterior adductor muscle (Fig. 5A). Anterior adductor muscle elongated, two times longer than posterior adductor, slightly curved parallel to anterodorsal shell margin, dorsal part narrower than ventral part. Posterior adductor muscle small, oval. Ctenidium thin, narrow, consisting of a single inner demibranch with fully reflected filaments (up to 50 filaments in large specimens more than 4.7 mm in length). Demibranch not covering lateral body pouches and consisting of both ascending and descending lamellae; ascending lamellae slightly shorter than descending lamellae. Labial palps small (to 150 µm), triangular, located at proximal end of longer (more than 1 mm) oral grooves extending to antero-ventral corner of gill (Figs. 5B–5F). Lateral pouches large (Figs. 5B, 5C, 5J, 5M and 5N), dorsoventrally elongated, oval in outline, with undulated margins and a few small, slightly projecting lobes along different margins; each pouch connecting to body by a wide neck (Figs. 5D–5F). Kidneys large, elongated, occupying a posterodorsal position between posterior adductor muscle and heart, containing numerous, bright orange, large (to 80 µm in diameter), different-size granules (Figs. 5D, 5J and 5H). Gonad occupying inner side of lateral pouches. Sexes are separate. Eggs oval or polygonal (up to 120–130 µm in length after fixation) (Fig. 5L). Alimentary system with short esophagus leading to a relatively large, elongate stomach; combined style sac and strongly curved midgut forming a deep and narrow loop between neck of lateral pouches and kidney; hind gut forming an anterior, deep, and wide loop dorsal to style sac, running posteriorly dorsal to kidney and posterior adductor muscle, opening at ventral side of posterior adductor muscle (Figs. 5I and 5P). Foot long, vermiform, depending upon its state of contraction, may either form coil within mantle cavity or form a relatively short, curved steam with bulbous tip; surface of bulbous portion with densely spaced papillae (Figs. 5D, 5K and 5N). Heel absent. Anterior and posterior pedal retractors short, narrow (Figs. 5D and 5F).

**Variability:** The shell shape and proportions, the degree of slope of anterodorsal and posterodorsal shell margins and curving of all shell margins vary significantly among different sized specimens (Figs. 2–4 and Table 2). Some specimens have a shell rather elongated dorsoventrally and ovate in outline. In some specimens, the shell is more rounded and angulate, the anterodorsal and posterodorsal margins are sloped more gently from beaks, the anterodorsal margin is more convex and the posterodorsal margin is almost straight, the ventral margin is more curved and slightly drawn out anteriorly. Moreover, in thicker-shelled specimens the hinge plate is wider and the inner thickenings of the shell in the аdductor muscle scar areas are much thicker and more expressed compared with specimens with a thinner shell (Figs. 4I, 4M, 4R and 4T).

**Distribution:** This species was recorded on the oceanic slope of the Kuril Islands (45°01.202′N, 151°06.008′E–47°14.32′N, 154°42.26′E) at a depth of 4,982–5,766 m, on the abyssal plain adjacent to the Kuril-Kamchatka Trench (39°43.43′N, 147°09.98′E–46°13.99′N, 155°33.10′E) at a depth of 4,860–5,497 m (bottom temperature (6–8 m above bottom) 1.5–1.6 °C, salinity 34.7‰, oxygen 7.71–7.72 ml. l^−1^) (Brandt et al., 2015), in the Kuril-Kamchatka Trench (44°06.152′N, 151°25.705′E–45°56.821′N, 152°51.185′E) at a depth of 6,068–9,540 m, and in the Japan Trench (36°44′N, 143°19′E–38°38′N, 144°06′E) at a depth of 7,350–7,540 m.

**Comparisons:** *Axinulus krylovae* sp. nov. strongly differs from all species of the genus *Axinulus* in having expressed thickenings of the shell in the adductor muscle scar areas, which are visible on the outer surface of the anterior and posterior parts of the shell as wide radial rays extending from the beaks (Table 3). Due to the shell thickening, the muscle scars are raised to various degrees above the inner shell surface, depending on the thickness of the shell itself. Also, *Axinulus krylovae* sp. nov. is clearly distinguished from all the other species by a unique sculpture of the prodissoconch (Table 3). Moreover, the new species well differs from almost all species of the genus *Axinulus* in having a conspicuous radial sculpture of the shell. Judging by photos of the type material and descriptions of species, *A. brevis*, *A. croulensis*, and *A. philippinensis* also have a radial sculpture on the shell surface, but it is less distinct and was described as fine radial lines or a peculiar radial texture (Oliver & Killeen, 2002; Allen, 2015). In contrast to most species of the genus, the new species has a larger shell. Only *A. oliveri* and *A. roseus* also have a large shell, but unlike *Axinulus krylovae* sp. nov., their shells lack the radial sculpture and inner thickenings in the adductor muscle scar areas and are characterized by a well defined escutcheon (Kamenev, 2020). In addition, the above species have extensively lobed lateral pouches.

**Table 3:** Main differentiating characters of *Axinulus* species.

Species	Maximum shell length and height (mm)	Shell	Sculpture	Escutcheon	Auricle	Lunule	Ligament	Lateral pouch	Prodissoconch length (µm) and sculpture	References
*Axinulus krylovae* sp. nov.	*L* = 5.2 *H* = 5.7	Elongated ovate to rhomboidal; anterodorsal and posterodorsal shell margins convex	Closely spaced, commarginal riblets with conspicuous radial ribs and rays and weak, irregular, undulations	Absent	Absent	Weakly defined, as a crest, raised, long, wide	Slightly visible externally as a narrow strip between valves	Large, no marked lobes	131–158; initial part with tubercle and depression, sculptured numerous, short folds and radial, long folds	Present study
*Axinulus alatus* sp. nov.	*L* = 2.7 *H* = 3.1	Oval to ovate-polygonal; anterodorsal and posterodorsal shell margins convex	Closely spaced, commarginal riblets; micro-sculpture of densely spaced pits	Weakly defined, long, narrow	Present, distinct	Weakly defined, as a weak crest, short, narrow, lanceolate	Not visible externally	Small, no marked lobes	161–174; initial part with densely spaced, short folds and wrinkles and two series of commarginal, long folds	Present study
*Axinulus cristatus* sp. nov.	*L* = 3.6 *H* = 3.8	Pyriform; anterodorsal and posterodorsal shell margins convex	Closely spaced, commarginal ribs and narrow, closely spaced, radial rays formed by closely spaced, finest, short, concentric wrinkles	Absent	Absent	Weakly defined, with a weak crest, long, wide	Not visible externally	No data	127; with 24 thin, almost straight folds extending from a long, high crest, located in mid-line of prodissoconch	Present study
*Axinulus roseus*	*L* = 8.7 *H* = 9.3	Rhomboidal; anterodorsal shell margin concave; posterodorsal shell margin convex or straight	Conspicuous, commarginal, narrow ridges with sparse, thin, radial rays from microscopic concentric wrinkles and weak, irregular undulations; microsculpture of densely spaced pits	Well defined, very long, narrow	Absent	Well defined, sunken, long, wide	Visible externally	Large, extensively lobed	212–215; with an oblique, shallow, wide, elongated sulcus in anterior part	Kamenev (2020)
*Axinulus oliveri*	*L* = 5.7 *H* = 6.2	Ovate-rhomboidal; anterodorsal shell margin convex or straight; posterodorsal shell margin convex	Thin, commarginal ribs and weak, irregular undulations; microsculpture of densely spaced pits	Well defined, very long, narrow	Present, distinct	Weakly defined, with a weak crest, long, wide	Not visible externally	Large, extensively lobed	191–220; initial part with 9–12 thin, lamellated folds extending from short, plicate ridge, located in mid-line of prodissoconch	Kamenev (2020)
*Axinulus antarcticus*	*H* = 2.9	Subquadrate; anterodorsal shell margin horizontal, straight; posterodorsal shell margin straight	Thin, commarginal ribs	Weakly defined, long, narrow	Present, distinct	Weakly defined, short, wide	Visible externally	Small, no marked lobes	115; sculptured with numerous folds, radiating from a central axis	Zelaya (2010)
*Axinulus philippinensis*	*L* = 3.2	Oblique, angular; anterodorsal shell margin convex or straight; posterodorsal shell margin convex or straight	Fine, growth lines and marginal, radial lines	Absent	Absent	No data	No data	Markedly lobed at margins	No data	Allen (2015)
*Axinulus alleni*	*L* = 2.5	Subquadrate; anterodorsal shell margin straight or convex; posterodorsal shell margin convex	Very fine, growth lines	Well defined, long, narrow	Present, distinct	No data	Visible externally	Large, swollen, without lobes	No data	Carrozza (1981), Payne & Allen (1991)
*Axinulus brevis*	*L* = 2.70 *H* = 3.19	Upright oval outline; sometimes pyriform; anterodorsal and posterodorsal shell margin convex	Very fine, growth lines and thin, radial riblets	Weakly defined, no sunken	Absent	Weakly defined	Visible externally	Large, elongate, no marked lobes	135–175	Verrill & Bush (1898), Payne & Allen (1991)
*Axinulus croulensis*	*L* = 1.8 *H* = 2.0	Upright oval or almost circular outline; anterodorsal shell margin horizontal, straight; posterodorsal shell margin convex	Very fine, commarginal lines and growth stops, mostly glossy with a radial texture	Absent	Indistinct	Absent	Visible externally	Large, with small projecting lobes along dorsal margin	121–141	Payne & Allen (1991), Oliver & Killeen (2002)
*Axinulus subequatorius*	*L* = 3.3 *H* = 3.5	Pyriform; anterodorsal and posterodorsal shell margins convex	Fine, ill-defined growth lines	Present	Absent	No data	No data	With a number of small peripheral lobes	No data	Payne & Allen (1991)

**Note:**

*L*, shell length; *H*, shell height.

**Derivation of name:** The new species was named in honor of the well-known malacologist E.M. Krylova, who made a significant contribution to the study of the world fauna of the family Vesicomyiidae and the superfamily Cuspidarioidea and has always provided invaluable help in my research on the deep-sea bivalve fauna of the northern Pacific.

**Remarks:** *Axinulus krylovae* sp. nov. has more or less expressed inner thickenings of the shell, depending on the shell thickness, in the area of adductor muscle scars. As a result, the muscle scars are raised above the inner shell surface. The thickened shell area is, as a rule, more pronounced posteriorly than anteriorly. The presence of elevated adductor scars is a characteristic and main distinguishing feature of species of the genus *Genaxinus* Iredale, 1930 from other genera of the family Thyasiridae (Hedley, 1907; Iredale, 1930; Oliver & Killeen, 2002; Oliver & Levin, 2006; Oliver, 2015). Nevertheless, I think the new species should be assigned to the genus *Axinulus* but not to *Genaxinus*. In species of *Genaxinus*, only muscle scars themselves are raised. The new species has the thickened anterior and posterior parts of shell, which are larger in area than the muscle scars proper, and these thickened areas bear muscle scars. The degree of inner shell thickening significantly varies, thus influencing the degree of elevation of muscle scars above the inner shell surface. Unlike the species described herein, in species of the genus *Genaxinus*, muscle scars bear prominent concentric growth marks that were formed during migration of the muscle during growth. In the *Genaxinus* species, the muscle scars are white color, project from the inner shell surface, and are well visible through the shell from outside (Payne & Allen, 1991; Oliver & Killeen, 2002).

The thickenings of the shell in muscle scar areas analogous to those of *Axinulus krylovae* sp. nov. are also characteristic of other species of the Thyasiridae (*e.g*., *A. croulinensis*, *A. alleni*, *Mendicula ultima* (Payne & Allen, 1991)) (Payne & Allen, 1991; Oliver & Killeen, 2002). In these species, inner shell thickening is also more expressed in the posterior area of the shell. Most probably, thorough studies of small thyasirids using scanning electron microscopy will reveal analogous inner shell thickenings in some other species as well.

The new species described here has a wide geographic and vertical distribution in the northwestern Pacific Ocean. It was found in large numbers in many samples from this vast region. This allowed a study of both the age and individual variability of its shell morphology. On the whole, the shell shape and proportions vary greatly, irrespective of the sampling area and depth. Specimens found in different areas and at different depths were of different size and had both the rounded and dorsoventrally elongated shell shape. A thorough examination of the shell morphology of specimens from various parts of the species range showed that they have the same main morphological characters (shell sculpture, hinge plate morphology, inner shell thickenings in muscle scar areas, prodissoconch sculpture). I found no unique morphological and anatomical features characteristic of a group of specimens. Moreover, specimens with a transitional shape of the shell between elongated oval and almost rounded were found in samples. Therefore, I think that specimens with different shell shape belong to the same species. It should be noted that many species of the Thyasiridae, among them species of *Axinulus*, exhibit a large age and individual variability of the shell shape and proportions (Payne & Allen, 1991; Oliver & Killeen, 2002; Allen, 2015; Kamenev, 2020).

*Axinulus krylovae* sp. nov. was found in the Pacific Ocean in a wide depth range (about 5,000 m). An earlier study recorded three species of bivalves with a vertical distribution range of 4,000 to 7,000 m among the hadal fauna of the Kuril-Kamchatka Trench. In addition, a number of eurybathic species from various taxonomic groups have a vertical distribution range greater than 5,000–7,000 m (Kamenev, 2019). By all appearances, *A. krylovae* sp. nov. also belongs to the group of eurybathic species inhabiting a wide depth range in the abyssal and hadal zones of the Pacific Ocean.

**Figure 6:**
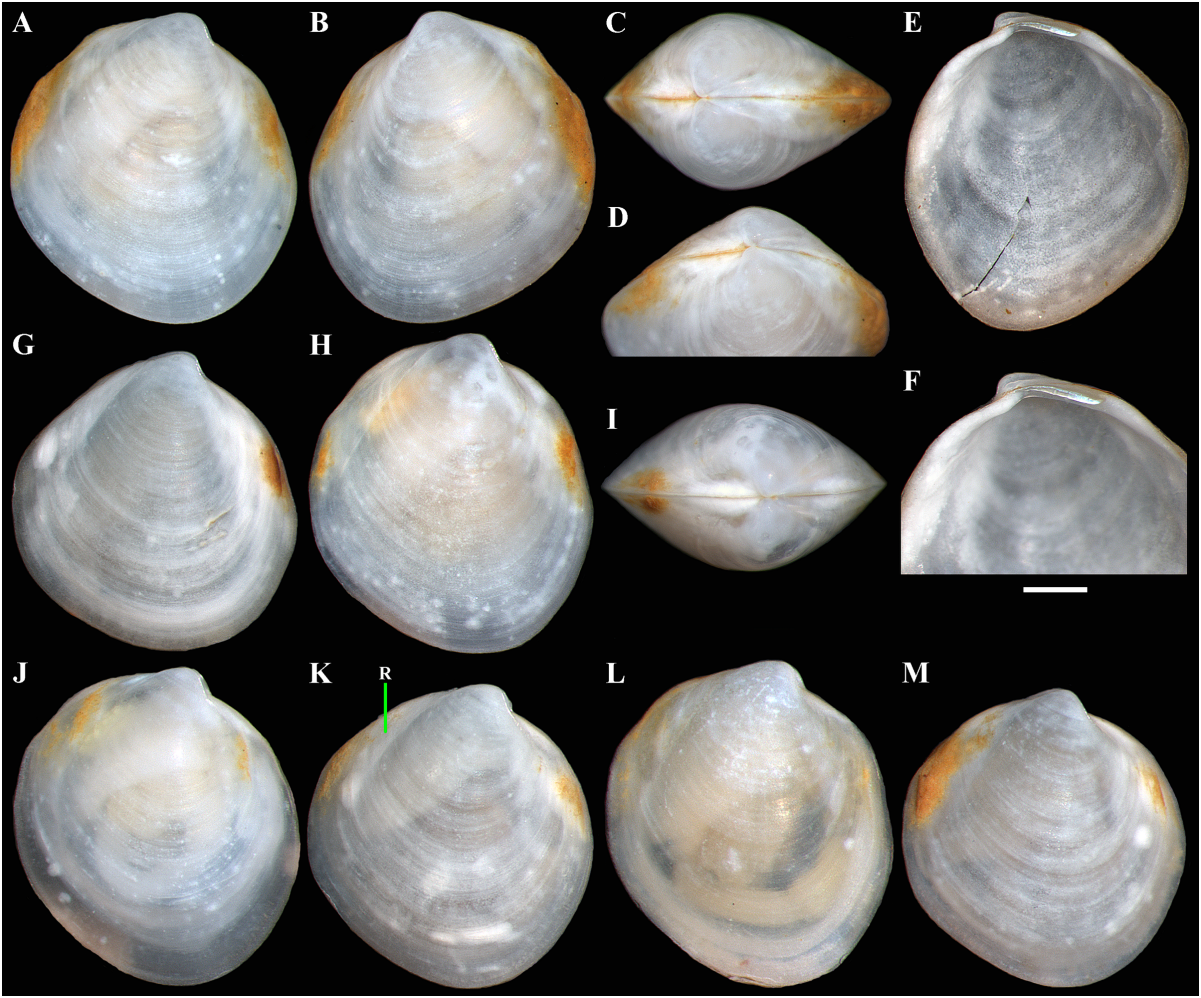
*Axinulus alatus* sp. nov. (A–D) Holotype ( MIMB 45893), exterior, dorsal, and oblique dorsal views of both valves, shell length 2.4 mm. (E and F) Interior view and hinge plate of right valve, valve length 2.7 mm. (G) Paratype (MIMB 45894), exterior view of right valve, shell length 2.7 mm. (H and I) Paratype (ZMF 372430), exterior view of right valve and dorsal view of both valves, shell length 2.3 mm. (J–M) Variability of shell shape, exterior view of right valves: (J) Shell length 1.7 mm; (K) Shell length 2.5 mm ; (L) Shell length 1.5 mm; (M) Shell length 2.1 mm. Abbreviation: R, radial, whitish ray. Scale bar: F = 500 µm.

**Figure 7:**
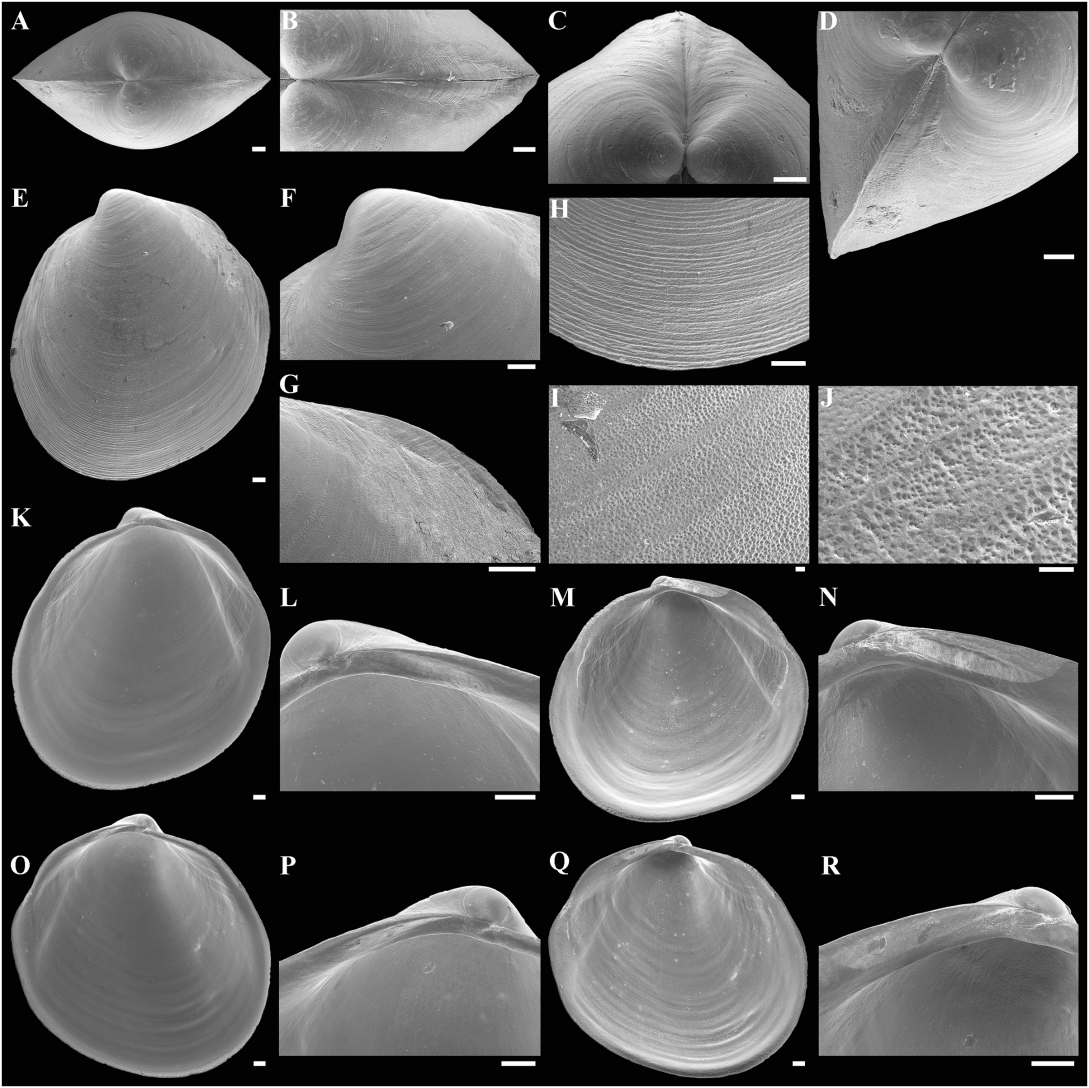
Scanning electron micrographs of *Axinulus alatus* sp. nov. (A) Dorsal view of both valves. (B and C) Escutcheon. (D) Lunule. (E) Exterior view of left valve. (F) Sculpture of beak region. (G) Posterodorsal margin of left valve with auricle. (H) Sculpture of ventral shell part. (I and J) Sculpture of central shell part. (K) Interior view of right valve. (L) Hinge plate and ligamental groove of right valve. (M and N) Ventral view of hinge plate and ligamental groove of right valve. (O) Interior view of left valve. (P) Hinge plate and ligamental groove of left valve. (Q and R) Ventral view of hinge plate and ligamental groove of left valve. Scale bars: A–H, K–R = 100 µm ; I, J = 10 µm.

**Figure 8:**
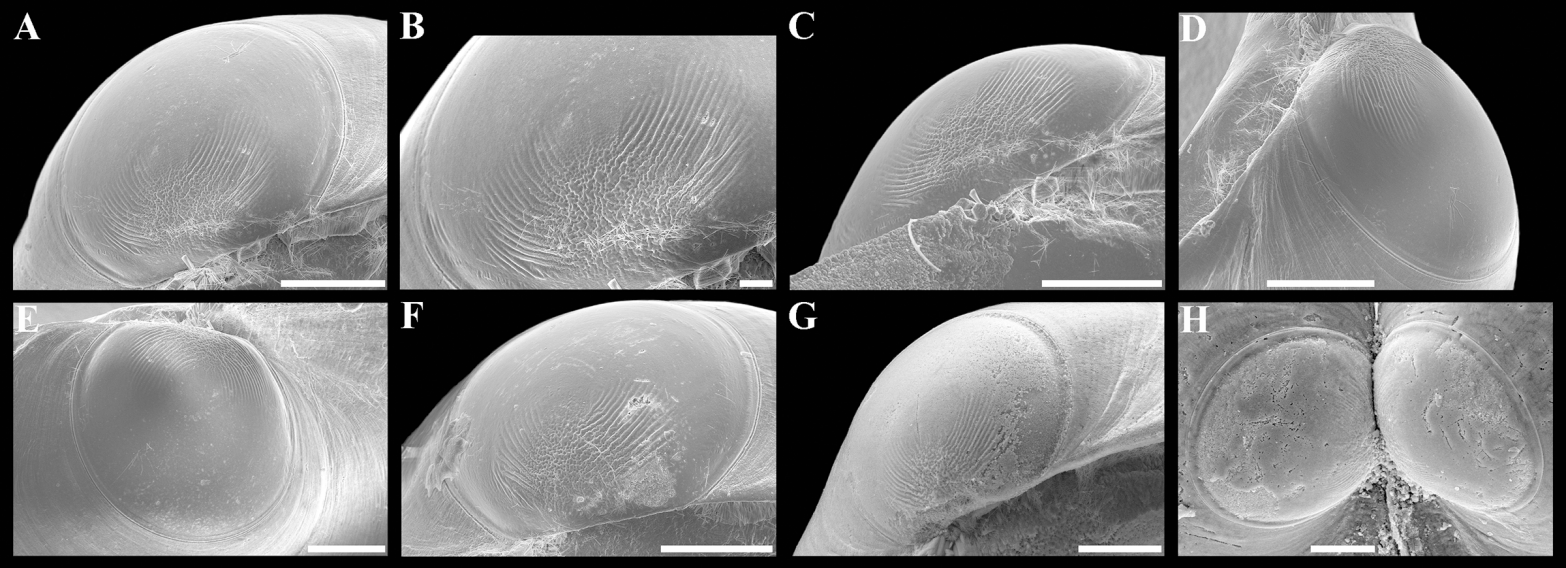
Scanning electron micrographs of prodissoconchs of *Axinulus alatus* sp. nov. (A–F) Prodissoconchs of specimens from oceanic slope of the Kuril Islands, depth 3,432 m. (G and H) Prodissoconchs of specimens from the Bering Sea, depth 3,978 m. Scale bars: A–H = 50 µm.

**Figure 9:**
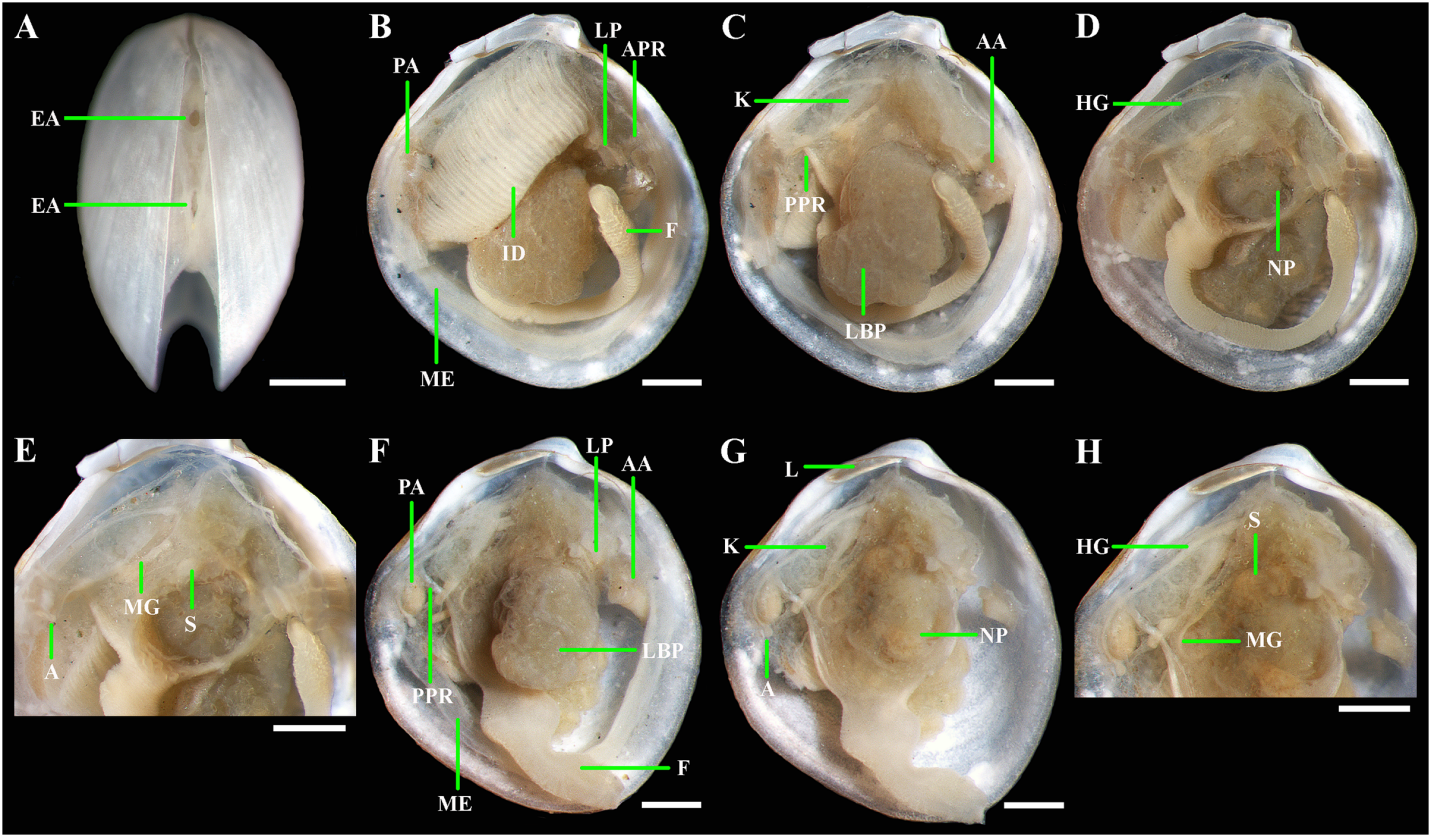
*Axinulus alatus* sp. nov. (A) Exhalant apertures. (B) Gross anatomy after removal of right valve and mantle. (C) Gross anatomy after further removal of right ctenidium. (D) Gross anatomy after further removal of right lateral body pouch. (E) Digestive system. (F) Gross anatomy after removal of right valve, mantle, right ctenidium, and bulbous portion of foot. (G) Gross anatomy after further removal of right lateral body pouch and mantle edge. (H) Digestive system. Abbreviations: AA, anterior adductor muscle; APR, anterior pedal retractor muscle; EA, exhalant aperture; F, foot; HG, hind gut; ID, inner demibranch; K, kidney; L, ligament; LBP, lateral body pouch; LP, labial palps; ME, mantle edge; MG, mid gut; NP, neck of lateral body pouch; PA, posterior adductor muscle; PPR, posterior pedal retractor muscle; S, stomach. Scale bars: A–H = 500 µm.

***Axinulus alatus* sp. nov.**

(Figs. 6–9, Tables 4 and 5)

**Table 4:** Additional material of *Axinulus alatus* sp. nov. examined in the present study.

Ship, cruise no.	Station	Date	Start		End		Depth (m)	Gear	*N*	Depository
			Latitude °N	Longitude °E	Latitude °N	Longitude °E				
**Abyssal slope of the Kuril Islands, Pacific Ocean**										
*Akademik M.A*. *Lavrentyev* 71	9-1	27.07.2015	46°16.082′	152°02.060′	–	–	3,432	GKG	10	MIMB 45895
	9-6	26.07.2015	46°16.103′	152°02.004′	46°16.132′	152°03.036′	3,436–3,377	EBS	2	MIMB 45896
	9-7	26.07.2015	46°16.129′	152°02.440′	46°16.070′	152°03.324′	3,409–3,377	EBS	1	MIMB 45897
**Commander Basin, Bering Sea**										
*Akademik Mstislav Keldysh* 22	2309	31.07.1990	55°13.2′	167°29.07′	55°12′	167°26.7′	3,957–3,978	ST	73	IORAS BIV00989
	2316	05.08.1990	55°36.1′	167°23.04′	55°35′	167°24.5′	4,294–4,200	ST	1	IORAS BIV00990
**Kodiak Island, Gulf of Alaska, Pacific Ocean**										
*Vityaz* 45	6095	08.05.1969	57°37.7′	148°34.5′ W	–	–	3,200	OG	2	IORAS BIV00991

**Note:**

OG, Okean grab (0.25 m^2^); GKG, giant box corer (0.25 m^2^); EBS, epibenthic sledge; ST, Sigsbee trawl; *N*, number of live specimens.

**Table 5:** *Axinulus alatus* sp. nov. Shell measurements (mm), indices and summary statistics of indices.

Depository	*L*	*H*	*A*	*W*	*H/L*	*A/L*	*W/L*
Paratype MIMB 45894	2.7	2.8	1.1	1.7	1.037	0.407	0.630
Paratype IORAS BIV00988	2.6	2.9	1.0	1.7	1.115	0.385	0.654
Paratype IORAS BIV00988	2.5	2.8	1.0	1.7	1.120	0.400	0.680
Paratype IORAS BIV00988	2.6	2.8	1.0	1.6	1.077	0.385	0.615
Paratype IORAS BIV00988	2.5	2.8	1.0	1.6	1.120	0.400	0.640
Paratype IORAS BIV00988	2.5	2.6	1.0	1.5	1.040	0.400	0.600
Holotype MIMB 45893	2.4	2.6	1.0	1.5	1.083	0.417	0.625
Paratype SMF 372430	2.3	2.6	0.9	1.4	1.130	0.391	0.609
IORAS BIV00989	2.4	2.6	0.9	1.5	1.083	0.375	0.625
IORAS BIV00989	2.3	2.5	0.8	1.4	1.087	0.348	0.609
IORAS BIV00989	2.2	2.4	0.8	1.4	1.091	0.364	0.636
IORAS BIV00989	2.2	2.4	0.9	1.3	1.091	0.409	0.591
IORAS BIV00989	2.2	2.4	0.8	1.3	1.091	0.364	0.591
IORAS BIV00989	2.1	2.3	0.7	1.3	1.095	0.333	0.619
IORAS BIV00989	2.1	2.3	0.8	1.3	1.095	0.381	0.619
IORAS BIV00989	2.1	2.5	0.7	1.2	1.190	0.333	0.571
IORAS BIV00989	2.1	2.3	0.7	1.3	1.095	0.333	0.619
IORAS BIV00989	2.0	2.4	0.7	1.4	1.200	0.350	0.700
IORAS BIV00989	2.0	2.2	0.9	1.3	1.100	0.450	0.650
IORAS BIV00989	2.0	2.3	0.7	1.3	1.150	0.350	0.650
IORAS BIV00989	2.0	2.2	0.6	1.3	1.100	0.300	0.650
IORAS BIV00989	2.0	2.1	0.8	1.2	1.050	0.400	0.600
IORAS BIV00989	1.9	2.2	0.6	1.2	1.158	0.316	0.632
IORAS BIV00989	1.9	2.0	0.7	1.2	1.053	0.368	0.632
IORAS BIV00989	1.9	2.1	0.6	1.2	1.105	0.316	0.632
IORAS BIV00989	1.9	2.1	0.8	1.1	1.105	0.421	0.579
IORAS BIV00989	1.9	2.1	0.7	1.1	1.105	0.368	0.579
IORAS BIV00989	1.9	2.1	0.8	1.1	1.105	0.421	0.579
IORAS BIV00989	1.8	2.0	0.7	1.1	1.111	0.389	0.611
IORAS BIV00989	1.7	1.9	0.5	1.0	1.118	0.294	0.588
IORAS BIV00989	1.6	1.8	0.5	0.9	1.125	0.313	0.563
IORAS BIV00989	1.5	1.7	0.5	0.9	1.133	0.333	0.600
Statistics	*L*	*H*	*A*	*W*	*H/L*	*A/L*	*W/L*
Mean	–	–	–	–	1.105	0.369	0.618
SE	–	–	–	–	0.026	0.033	0.024
SD	–	–	–	–	0.037	0.039	0.031
Min	–	–	–	–	1.037	0.294	0.563
Max	–	–	–	–	1.200	0.450	0.700

**Note:**

*L*, shell length; *H*, height; *W*, width; *A*, anterior end length.

*Axinulus* sp.: *Kamenev*, 2018a, p. 234.

urn:lsid:zoobank.org:act:0BBDAC37-2258-4C8B-89E4-DECF7402C58E

**Type material and locality:** Holotype (MIMB 45893), slope of the Kuril Islands, Pacific Ocean (46°16.082′N, 152°02.060′E), 3,432 m, boxcorer, Coll. G.M. Kamenev, 27-VII-2015 (RV *Akademik M.A. Lavrentyev*, cruise no. 71, stn. 9-1); paratype (SMF 372430), from holotype locality; paratypes (5) (IORAS BIV00988) and paratype (MIMB 45894), Commander Basin, Bering Sea (55°13.2′N, 167°29.07′E–55°12′N, 167°26.7′E), 3,957–3,978 m, Sigsbee trawl, Coll. S.V. Galkin, 31-VII-1990 (RV *Akademik Mstislav Keldysh*, cruise no. 22, stn. 2309).

**Other material examined:** 89 live specimens (Table 4).

**Diagnosis:** Shell small (to 2.9 mm in length), oval to ovate-polygonal, slightly drawn out anterior, with two slightly curved, radiating, whitish, elongate-triangular, opaque rays extend from beaks to anteroventral and posteroventral margins. Sculpture of closely spaced, commarginal riblets. Micro-sculpture of densely spaced pits. Posterior folds and sulcus absent. Escutcheon long, narrow, shallow. Auricle long, low. Lunule as a weak crest, slightly raised, short, lanceolate. Ligament sunken, not visible externally, short. Prodissoconch medium in size (length 161–174 µm); initial part with small area of densely spaced, short, curved folds and wrinkles and two series of commarginal, long, thin folds extending as wings from this area. Adductor muscle scars distinct, elongated triangular in outline; posterior scar raised above inner shell surface due to thickening of shell. Lateral body pouches small, simple, without projecting lobes.

**Description.** Shell small (to 2.9 mm in length and 3.1 mm in height), inflated (W/L = 0.618 ± 0.024), higher than long (H/L = 1.105 ± 0.026), slightly drawn out anteriorly, median area divided by a weak change in angulation; oval to ovate-polygonal, sometimes slightly pyriform, subequilateral, white, thin, translucent, with two slightly curved, radiating, whitish, elongate-triangular, opaque rays extending from beaks to anteroventral and posteroventral margins, respectively, more marked in posterior shell area, formed by internal thickening of shell associated with anterior and posterior adductor muscles; patches of silty deposit adhering to anterior and posterior shell margins (Fig. 6 and Table 5). Periostracum very thin, colorless, translucent, adherent. Dissoconch sculptured with thin, closely spaced, commarginal riblets. Micro-sculpture of small, densely spaced pits. Beaks small, prosogyrate, curved slightly inwards, anterior to midline (A/L = 0.369 ± 0.033) (Fig. 7 and Table 5). Anterodorsal shell margin long, convex, steeply sloping from beaks, forming a rounded angle at transition to anterior margin. Anterior shell margin curved, smoothly transitioning to ventral margin. Ventral margin strongly curved, slightly angulate. Posterodorsal shell margin long, convex, steeply sloping from beaks, forming a broadly rounded angle at transition to posterior margin. Posterior shell margin slightly curved, smoothly transitioning to ventral margin. Posterior folds absent but posterior shell area a little flattened. Escutcheon long, almost as long as entire length of posterodorsal shell margin, narrow, shallow, demarcated by low ridges. Auricle long, almost as long as entire length of escutcheon, weak, low, only slightly projecting (Figs. 7B, 7C and 7G). Lunule as a weak crest, slightly raised, short, about half length of anterodorsal shell margin, narrow, lanceolate, weakly defined by low and thin ribs (Fig. 7D). Ligament opisthodetic, sunken, not visible externally, short, about one third of entire length of the escutcheon, thick, almost straight, lying in deep, wide groove in hinge plate (Figs. 7L–7N, 7P–7R). Prodissoconch medium in size (length 161–174 µm), distinctly separated from dissoconch, ovate in outline. Initial part of prodissoconch with densely spaced, short, curved folds and wrinkles interspersed with pits, forming a rounded zone. This zone giving rise to two series of commarginal, long, thin, low folds (about 20 folds in each series), extending as wings in different directions; remaining surface of prodissoconch smooth (Fig. 8). Hinge plate thin, edentulous, with almost straight ligamental groove (Figs. 7L and 7P). Adductor muscle scars distinct, long, elongated triangular in outline, extending into umbonal cavity, bearing thin, radial striation. Posterior adductor scar narrow, straight, raised above inner shell surface due to thickening of shell in this area. Anterior adductor scar wider than posterior one, curved, not raised above inner shell surface (Figs. 7K, 7M, 7O and 7Q).

*Anatomy:* Mantle thin; margins thickened, unfused except long posterior suture with two exhalant apertures below the posterior adductor (Fig. 9A). Anterior adductor muscle elongated, two times longer than posterior adductor, slightly curved, dorsal part narrower than ventral part. Posterior adductor muscle small, oval (Figs. 9B and 9C). Ctenidium thin, narrow, consisting of a single inner demibranch with fully reflected filaments (up to 30 filaments in largest specimen 2.9 mm in shell length). Demibranch not covering lateral body pouches and consisting of both ascending and descending lamellae; ascending lamellae slightly shorter than descending lamellae. Labial palps small, as extensions of the short (about 300 µm), oral grooves (Fig. 9B). Lateral body pouches small (Figs. 9C and 9F), dorsoventrally elongated, outline oval, with undulated margins, without projecting lobes; each pouch connecting to body by a wide neck (Fig. 9D). Kidneys large, dorsoventrally elongated along posterodorsal shell margin, without granules (Figs. 7C–7G). Alimentary system with short oesoghagus leading to an elongate stomach; combined style sac and midgut strongly curved, forming a deep and narrow loop between neck of lateral pouches and kidney; hind gut forming an anterior, deep, and wide loop dorsal to style sac, running posteriorly dorsal to kidney and posterior adductor muscle, opening at ventral side of posterior adductor muscle (Figs. 7E and 7H). Foot long, vermiform, distally bulbous, with a muscular ring at the junction with the visceral mass. Bulbous portion of foot not divided into two distinct parts; surface with densely spaced papillae; heel absent. Anterior and posterior pedal retractors short, narrow (Figs. 7B, 7C and 7F).

**Variability:** The shell shape and proportions vary little among different-sized specimens (Figs. 6G–6M and Table 5). The shell shape is oval to ovate. Some specimens have a more angular and relatively shortened shell. In small specimens (less than 2 mm in shell length), the shell is more elongated dorsoventrally, with a more anteriorly drown-out ventral margin.

**Distribution:** This species was recorded on the oceanic slope of the Kuril Islands (46°16.082′N, 152°02.060′E–46°16.129′N, 152°03.324′E) at a depth of 3,377–3,436 m, at the bottom of Commander Basin (Bering Sea) (55°12.7′N, 167°26.7′E–55°36.1′N, 167°23.04′E) at a depth of 3,957–4,294 m, and off Kodiak Island (Gulf of Alaska, Pacific Ocean) (57°37.7′N, 148°34.5′W) at a depth of 3,200 m.

**Comparisons:** *Axinulus alatus* sp. nov. differs from all species of the genus *Axinulus* in having a unique sculpture of the prodissoconch (Table 3). In terms of the shape, proportions, and size of the shell, *A. alatus* sp. nov. is closest to *A. brevis*, *A. croulensis*, *A. antarcticus*, and *A. subequatorius*. However, unlike *A. brevis* and *A. croulensis*, the new species described here lacks radial sculpture of the shell and has a well-defined, long auricle, and a deep sunken ligament invisible externally. Apart from the differences in prodissoconch sculpture, *A. alatus* sp. nov. is distinguished from *A. antarcticus* by having a long lanceolate lunule and a deep sunken ligament invisible externally. *Axinulus alatus* sp. nov. differs from *A. subequatorius* in lacking projecting lobes of lateral body pouches and in having two posterior inhalant apertures but not one (Payne & Allen, 1991). *Axinulus alatus* sp. nov. is similar in shell shape and proportions to some species of the genus *Adontorhina* Berry, 1947, but it is readily distinguished from them by the absence of irregular, minute granules on the hinge plate (Scott, 1986; Kamenev, 1996, 2013; Coan, Scott & Bernard, 2000; Güller & Zelaya, 2011; Coan & Valentich-Scott, 2012; Valentich-Scott, Coan & Zelaya, 2020).

**Derivation of name:** The species epithet meaning “winged” indicates the similarity of two series of long, commarginal folds of the prodissoconch to the extended wings of a flying bird.

**Remarks:** *Axinulus alatus* sp. nov. has a wide range in the northern Pacific Ocean and was found off the Asian and American continents in the lower abyssal zone in the depth range of 3,000–5,000 m. The species was not found among extensive material of bivalves collected by the numerous deep-sea expeditions at depths greater than 5,000 m at the abyssal plain adjacent to the Kuril-Kamchatka and Japan trenches (Okutani, 1974, 2000; Okutani & Fujikura, 2002; Okutani & Kawamura, 2002; Kamenev, 2015, 2018a), as well as in the hadal zone of the Aleutian, Kuril-Kamchatka, and Japan trenches (Okutani, 1974, 2003; Okutani, Fujikura & Kojima, 1999; Fujikura et al., 1999, 2002; Okutani & Fujiwara, 2005; Kamenev, 2019, 2020, 2023). In addition, this species was not recorded for the benthic fauna of the Kuril Basin (the Sea of Okhotsk) a little less than 3,300 m deep (Kamenev, 2018a), though it was found on the oceanic slope of the Kuril Islands opposite the deepest Bussol Strait connecting the Sea of Okhotsk and the Pacific Ocean. The deep-sea ecosystem of the Kuril Basin is characterized by low oxygen concentrations near the bottom, largely influencing the composition and quantitative distribution of benthic fauna (Kamenev et al., 2022). It is possible that oxygen deficiency exerts a negative influence, not allowing the species to live in the Kuril Basin. In the Commander Basin (Bering Sea), in contrast to the Kuril Basin, *A. alatus* sp. nov. forms high-density populations. Probably, *A. alatus* sp. nov. is a low abyssal species, preferring depths of 3,000–5,000 m.

***Axinulus cristatus* sp. nov.**

(Fig. 10)

**Figure 10:**
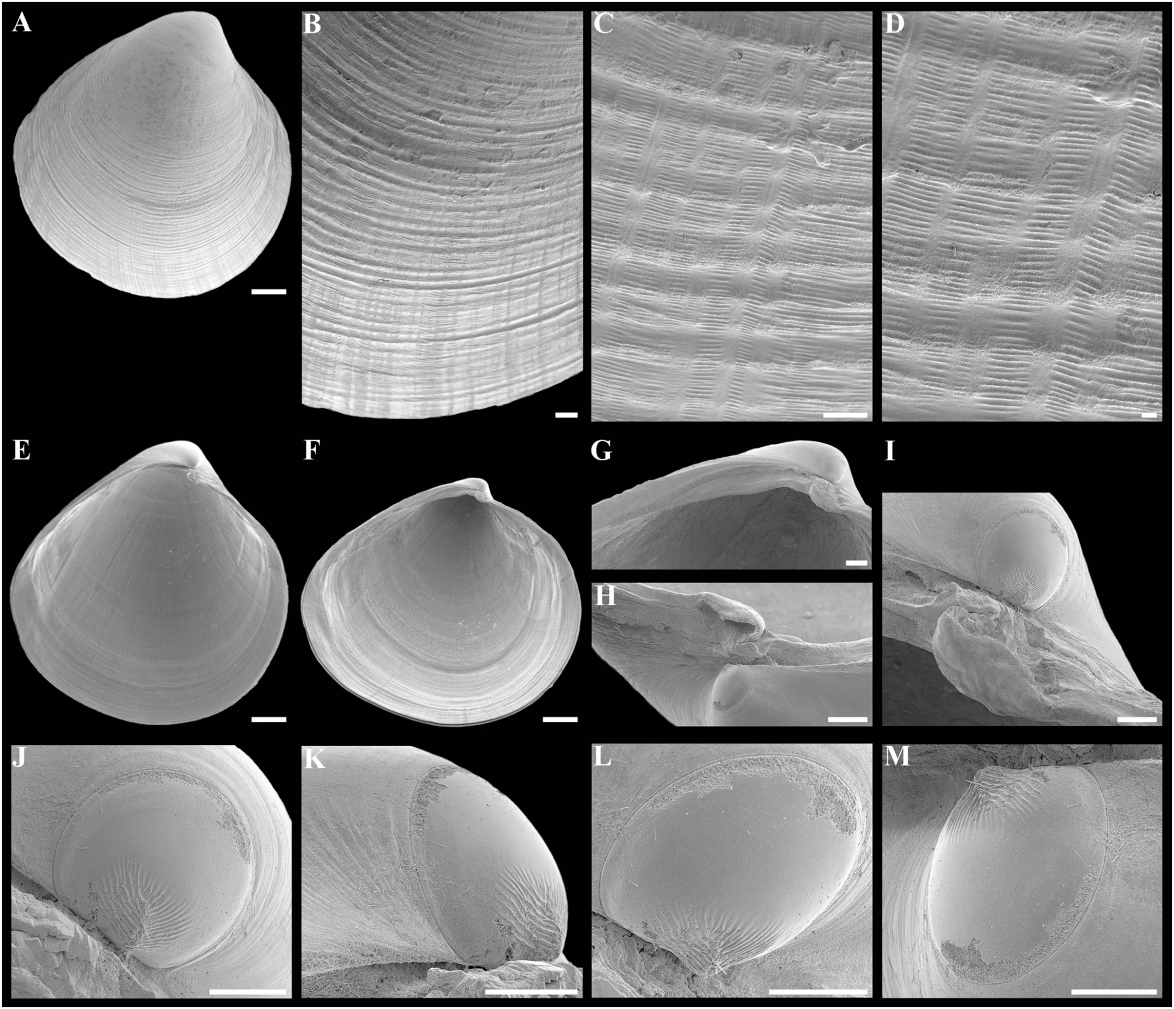
Scanning electron micrographs of the holotype of *Axinulus cristatus* sp. nov. (MIMB 45892). (A) Exterior view of right valve. (B–D) Sculpture of ventral shell part. (E) Interior view of left valve. (F) Ventral view of left valve. (G) Hinge plate and ligamental groove of left valve. (H and I) Spoon-shaped tubercle under beak in left valve. (J–M) Prodissoconch. Scale bars: A, E, F = 500 µm; B, G, H = 100 µm; C, I, J–M = 50 µm; D = 10 µm.

urn:lsid:zoobank.org:act:BFDA7E99-6561-4597-B5BE-5037F42815B0

**Type material and locality:** Holotype (MIMB 45892), Kuril-Kamchatka Trench, Pacific Ocean (45°56.821′N, 152°51.185′E – 45°56.834′N, 152°50.943′E), 6,168–6,164 m, epibenthic sledge, Coll. A. Brandt, 27-VIII-2016 (RV *Sonne*, cruise no. 250, stn. 30).

**Diagnosis:** Shell medium in size (to 3.6 mm in length), pyriform. Sculpture of closely spaced, commarginal ribs and narrow, radial rays consisting of fin, short, concentric wrinkles. Posterior folds and sulcus absent. Escutcheon and auricle absent. Lunule as a weak crest, raised, long, wide, weakly defined. Ligament sunken, long. Prodissoconch small (length 127 µm), with 24 thin, almost straight folds of different length, extending from high crest, located in mid-line of prodissoconch. Adductor muscle scars distinct, elongated triangular in outline, not raised above inner surface of shell. Inner shell surface with distinct, thin, radial striae.

**Description:** Shell medium in size (to 3.6 mm in length and 3.8 mm in height), pyriform, equivalve, subequilateral, white, thick, inflated, slightly higher than long (H/L = 1.043) (Fig. 10). Periostracum thin, colorless, translucent, adherent. Dissoconch sculptured with thin, closely spaced, commarginal ribs and narrow, closely spaced, radial rays of varying width, formed by closely spaced, finest, short, concentric wrinkles; radial rays becoming widest and prominent along shell margins (Figs. 10B–10D). Beaks small, raised, prosogyrate, anterior to midline (A/L = 0.312). Anterodorsal shell margin convex, steeply sloping from beaks, smoothly transitioning to anterior margin. Anterior margin curved, smoothly transitioning to ventral margin. Ventral margin gently curved. Posterodorsal margin long, slightly convex, steeply sloping from beaks, descending to mid-point of shell, forming a rounded angle at transition to slightly curved posterior margin. Posterior folds and sulcus absent. Escutcheon and auricle absent. Lunule as a weak crest, raised, long, wide, weakly defined, demarcated by weak, long, rounded ridges along entire anterodorsal shell margin. Ligament opisthodetic, sunken, thick, evenly curved, long, about half the length of posterodorsal shell margin, lying in deep, curved groove at surface of hinge plate (Figs. 10E–10G). Prodissoconch small (length 127 µm), distinct, oval in outline, convex, with 24 thin, almost straight folds of different length, sometimes bifurcated at end, extending from a long, high crest, located in mid-line of prodissoconch; remaining surface of prodissoconch smooth (Figs. 10J–10M). Hinge plate thickened, with a distinct, flattened, spoon-shaped tubercle under beak in left valve and a long ligamental groove (Figs. 10G–10I). Adductor muscle scars distinct, long, outline elongated triangular, extending into umbonal cavity, bearing thin, indistinct, radial striation, not raised above shell surface. Posterior adductor scar narrow, straight. Anterior adductor scar about two times wider than posterior and curved. Inner shell surface with distinct, thin, radial lines, corresponding to radial sculpture of external shell surface (Figs. 10E and 10F).

**Distribution:** This species is known only from the holotype locality in the Kuril-Kamchatka Trench at a depth of 6,168–6,164 m.

**Comparisons:** *Axinulus cristatus* sp. nov. is readily distinguished from all species of the genus *Axinulus* in having a radial sculpture in the form of rays consisting of closely spaced, short, fin, wrinkles and distinct radial lines on the inner shell surface, as well as by its unique sculpture of the prodissococh (Table 3). In addition, the new species differs from other species of the genus *Axinulus* in having a distinct spoon-shaped tubercle on the hinge plate in the left valve. However, the unusual shape of the tubercle on the hinge plate may be an individual abnormality of the examined specimen rather than being a specific character.

**Derivation of name:** The species name means “crested” and refers to a high crest on the surface of the prodissoconch.

**Remarks:** Unfortunately, only a single specimen of *A. cristatus* sp. nov. was found after examining all the material collected by many expeditions in the northwestern Pacific Ocean. Therefore, the gross anatomy of the body of this species is not studied, because the body has partially dissolved during preparation of the specimen for examination in a scanning microscope. In the specimen described here, the gill had only one demibranch; therefore, I assigned the species to the genus *Axinulus*. Since the shell of the species has a number of unique morphological characters that readily separate it from the congeners, I described it as new to science, despite the minimal material at my disposal. Due to the difficulty of collecting bottom animals from great depths, many deep-sea species are often represented by a single specimen in the benthic fauna collections made by different deep-sea expeditions (Kamenev, 2015, 2019) and are thus described from merely 1–2 specimens (*e.g*., Knudsen, 1970; Payne & Allen, 1991).

## Discussion

Until recently, the genus *Axinulus* was represented only by six species (Zelaya, 2010; Allen, 2015; Oliver, 2015; Kamenev, 2020). Of these, five species were found in the Atlantic Ocean (Payne & Allen, 1991; Allen, 2008; Zelaya, 2010), while in the Pacific Ocean only one species of the genus, *A. philippinensis*, was recorded and described from the bottom of the Philippine Trench (Allen, 2015). In recent years, a study of the extensive material of bivalves collected by many deep-sea expeditions mainly in the northwestern Pacific Ocean, another five species of the genus *Axinulus* were recorded for the Pacific Ocean (Kamenev, 2020). Thus, the number of species in the genus has increased almost twice, and at present the fauna of *Axinulus* in the Pacific Ocean has more species, compared to the Atlantic Ocean.

Out of all the species found in the Atlantic Ocean, only *A. subequatorius* was recorded exclusively in the abyssal zone at depths of more than 3,000 m. The other species have a wider vertical distribution range and were recorded in the subtidal and bathyal zones at depths less than 3,000 m. In contrast to Atlantic species, all Pacific species of the genus *Axinulus* were found exclusively at depths greater than 3,000 m. Moreover, almost all of them, except *A. alatus* sp. nov., were recorded in the hadal zone of different oceanic trenches at depths of more than 6,000 m (Allen, 2015; Kamenev, 2020). Thus, unlike the Atlantic Ocean, species of the genus *Axinulus* are typical members of the abyssal and hadal benthic fauna in the northern Pacific Ocean. In addition, the species *A. roseus*, *A. krylovae* sp. nov., *A. philippinensis*, and *A. alatus* sp. nov. were found in large numbers in samples collected from the floor of the deepest basins of the Kuril-Kamchatka, Japan, and Philippine trenches, and the Bering Sea, respectively (Allen, 2015; Kamenev, 2020; Kamenev et al., 2022), where they probably play a substantial role in the functioning of benthic deep-sea ecosystems in these northwestern Pacific regions.

Presumably, species of the genus *Axinulus* were found in another seven oceanic trenches, apart from the Kuril-Kamchatka, Japan, and Philippine trenches (Belyaev, 1989). Therefore, further study will most likely increase the species richness of the genus *Axinulus*.

Unlike many species of the genus *Thyasira*, many species of the genus *Axinulus* have a uniquely sculptured prodissoconch, which is one of the important diagnostic characters of thyasirids (Zelaya, 2009, 2010; Kamenev, 2020, 2023). The prodissoconch sculpture of *A. brevis*, *A. alleni*, *A. subequatorius*, and *A. philippinensis* has not been reported. Likewise, the prodissoconch of *A. croulensis*, which is smooth and lacks sculpture, was not examined with a scanning microscope (Oliver & Killeen, 2002). Perhaps, the prodissoconch of all the above species has an external sculpture that can be distinguished only at high magnification using scanning microscopy. It cannot be ruled out that further examination of the prodissoconch of these species will reveal more morphological features, allowing a more accurate diagnosis of largely morphologically similar species of the genus *Axinulus*.

## Additional Information and Declarations

### Data Availability

The following information was supplied regarding data availability: The raw data is available in Figs. 2–10 and in Tables 1–5. The type and other specimens of all new species examined in this study are deposited in the Museum of A.V. Zhirmunsky National Scientific Center of Marine Biology, Far Eastern Branch, Russian Academy of Sciences, Vladivostok (Accessions: MIMB 45893-45960), P.P. Shirshov Institute of Oceanology, Russian Academy of Sciences, Moscow (Accessions: IORAS BIV00980-00992), and Senkenberg Museum Frankfurt (Accessions: SMF 372429, 372430).

### New Species Registration

The following information was supplied regarding the registration of a newly described species: Publication LSID: urn:lsid:zoobank.org:pub:0AE8C4E1-0F13-478E-81E9-287980139049 *Axinulus krylovae* sp. nov. LSID: urn:lsid:zoobank.org:act:F3D588B6-84FD-44A7-99BF-7FB53AC893F3 *Axinulus alatus* sp. nov. LSID: urn:lsid:zoobank.org:act:0BBDAC37-2258-4C8B-89E4-DECF7402C58E *Axinulus cristatus* sp. nov. LSID: urn:lsid:zoobank.org:act:BFDA7E99-6561-4597-B5BE-5037F42815B0
